# Data-driven deformation correction in X-ray spectro-tomography with implicit neural networks

**DOI:** 10.1016/j.patter.2026.101515

**Published:** 2026-03-30

**Authors:** Ting Wang, Zipei Yan, Hongyi Pan, Kai Zhang, Michael K.-P. Ng, Xiqian Yu, Chao Wang, Jizhou Li

**Affiliations:** 1 Department of Statistics and Data Science, Southern University of Science and Technology, Shenzhen, China; 2 Department of Electronic Engineering, The Chinese University of Hong Kong, Hong Kong, China; 3 Beijing National Laboratory for Condensed Matter Physics, Institute of Physics, Chinese Academy of Sciences, Beijing, China; 4 Beijing Synchrotron Radiation Facility, X-ray Optics and Technology Laboratory, Institute of High Energy Physics, Chinese Academy of Sciences, Beijing, China; 5 Department of Mathematics, Hong Kong Baptist University, Hong Kong, China; 6 CUHK Shenzhen Research Institute, Shenzhen, China; 7 Shun Hing Institute of Advanced Engineering, The Chinese University of Hong Kong, Hong Kong, China

**Keywords:** X-ray spectro-tomography, deformation correction, self-supervised learning, coordinate-based neural network, implicit neural representations

## Abstract

Full-field transmission X-ray microscopy with X-ray absorption near-edge structure spectroscopy enables non-destructive, high-resolution, chemically specific three-dimensional morphological and compositional analyses. However, spectro-tomographic acquisitions often suffer from image deformations and misalignments caused by mechanical instabilities and hardware limitations, which can substantially degrade the quality of tomographic reconstruction and downstream analyses. This critical bottleneck hinders the broader application of X-ray spectro-tomography in addressing complex scientific problems across various disciplines. To address this, we introduce CANet, a self-supervised coordinate-based neural network that implicitly models deformation fields to efficiently and accurately correct misalignment. Unlike traditional methods, CANet requires no external training data and learns a continuous mapping from projection spectral or angular coordinates to affine transformations, enabling unified registration across both tomographic and spectral dimensions. Demonstrated on X-ray spectro-tomographic datasets of battery cathode particles, CANet achieves robust alignment and restores high-fidelity structural and chemical contrast, thereby facilitating the resolution of nanoscale degradation mechanisms.

## Highlights

• CANet is a self-supervised method correcting X-ray spectro-tomography deformations
• Coordinate-based network models continuous deformation for precise image alignment
• The method unifies tomographic and spectral alignment in a single framework
• Robust alignment enables high-fidelity structural and chemical imaging contrast

## The bigger picture

Understanding the complex interplay between structure and chemistry at the nanoscale is pivotal for advancing energy storage technologies. High-resolution X-ray spectro-tomography offers powerful insights into these 3D chemical states, yet its potential is often limited by experimental instabilities that cause image misalignment and deformation. This work presents a data-driven solution that bypasses the need for manual markers or extensive training datasets, offering a robust, automated path to high-fidelity chemical imaging. By effectively resolving nanoscale degradation features such as intragranular cracking and oxidation state heterogeneity, this approach empowers researchers to design more durable and efficient battery materials while also providing a versatile tool applicable to broader fields in materials science.

## Teaser

Wang et al. introduce CANet, a self-supervised, coordinate-based neural network designed to correct complex deformations in X-ray spectro-tomography. By implicitly modeling deformation fields without external training data, CANet achieves robust alignment across both tomographic and spectral dimensions. This framework significantly enhances the fidelity of 3D chemical mapping, enabling the precise visualization of nanoscale degradation mechanisms in battery materials.

## Introduction

The ability to characterize the three-dimensional (3D) structure and chemical composition of materials at the nanoscale is fundamental to advancing fields ranging from materials science and chemistry to biology and geology. Synchrotron-based X-ray microscopy techniques have emerged as indispensable tools for this purpose, offering non-destructive imaging with high spatial resolution and elemental sensitivity.^1^^,^^2^ Among these, full-field transmission X-ray microscopy (TXM) combined with X-ray absorption near-edge structure (XANES) spectroscopy, a technique often referred to as spectro-microscopy, provides two-dimensional (2D) maps of chemical states and elemental distributions.^3^^,^^4^^,^^5^^,^^6^^,^^7^ By acquiring a series of 2D projections at various sample rotation angles and applying tomographic reconstruction algorithms, this technique can be extended into 3D, a modality known as X-ray spectro-tomography. This four-dimensional (4D) imaging capability (3D spatial information combined with energy) enables comprehensive, quantitative analysis of morphological and chemical heterogeneity within intact samples.^6^^,^^8^^,^^9^ Furthermore, modern synchrotron facilities can further enhance this capability by providing high flux, spatial coherence, and monochromaticity, opening vast opportunities for chemical mapping and time-resolved studies.^10^

Spectro-tomography provides critical insights into energy storage, where material performance and degradation are governed by complex chemomechanical processes at the nanoscale.^4^^,^^6^^,^^11^ For instance, in lithium-ion batteries, *in operando* TXM-XANES has been indispensable for tracking phase transformations,^6^^,^^12^^,^^13^ mapping state-of-charge heterogeneity within and between electrode particles,^14^ and understanding the influence of local structure on ion diffusion.^15^ These studies have revealed that degradation mechanisms often originate from local heterogeneities, underscoring the necessity of high-resolution 3D chemical imaging to guide the design of next-generation battery materials.^16^^,^^17^

Despite its power and broad applicability, TXM-based spectro-tomography is frequently hampered by a significant technical challenge: image misalignment and deformation during data acquisition. A complete spectro-tomography dataset consists of hundreds to thousands of 2D projections, acquired over a range of rotation angles and X-ray energies. This process can take tens of minutes to hours, during which the sample’s position can drift. These misalignments and deformations arise from two primary sources. First, instrumental and energy-dependent changes cause global geometric offsets. Mechanical vibrations, stage/optics drift, and source fluctuations lead to image-wise translations.^18^^,^^19^ Additionally, switching X-ray energy necessitates focal adjustments along the optical axis. This changes the projection geometry, resulting in isotropic scaling differences between energy channels. Second, a particularly challenging issue for *operando* studies is that the sample itself can undergo intrinsic deformation. In battery electrodes, electrochemical cycling induces volume changes, particle movement, and electrode breathing, resulting in complex, non-rigid, and non-linear deformations.^20^ If uncorrected, these misalignments and deformations severely degrade the quality of the tomographic reconstruction, introducing significant artifacts, blurring fine features, and ultimately compromising the spatial resolution and quantitative accuracy of the resulting 3D chemical maps.^21^ This presents a critical bottleneck that limits the full potential of X-ray spectro-tomography.

**Figure 1:**
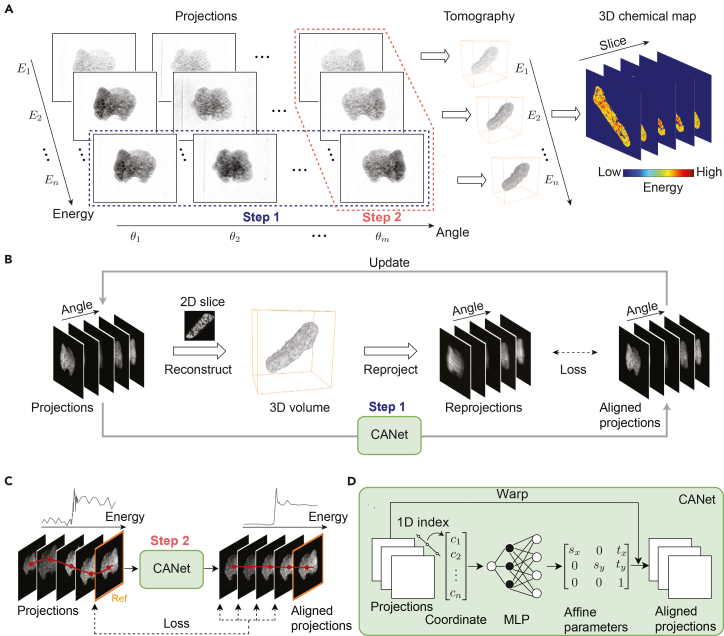
Overview of the proposed deformation correction method for X-ray spectro-tomography (A) The general processing workflow for sequential tomographic (step 1) and spectral (step 2) alignment. (B) Schematic of the iterative projection registration workflow for step 1 (tomographic alignment). (C) Schematics of the projection registration workflow for step 2 (spectral alignment). (D) The framework of the proposed CANet. This self-supervised architecture employs a multilayer perceptron (MLP) to update affine parameters. CANet minimizes the loss between aligned projections and reprojections (for tomography) or the highest-energy reference (for spectral).

To address this challenge, various image registration algorithms have been developed. As illustrated in Figure 1A, the registration process generally consists of two sequential steps: tomography and spectral alignment. In step 1 (tomography alignment), projections acquired at different angles for a single energy level (typically the highest energy) are aligned to serve as a baseline. There are three representative methods for this task. The first one utilizes fiducial markers or recognizable features within the sample for registration.^22^^,^^23^^,^^24^^,^^25^^,^^26^^,^^27^ While effective, this marker-based approach is cumbersome and impractical, as suitable features are not always available. The second approach employs capacitance sensors or laser interferometers to experimentally measure the rotation stage’s runout and correct offsets.^19^ However, this hardware-based approach is costly and sensitive to environmental factors, such as temperature and humidity. The third method, based on the concept of tomographic consistency,^28^ is the projection-reprojection correction method. This technique iteratively refines alignment by registering experimental projections against numerical “reprojections” generated from a preliminary 3D reconstruction.^29^^,^^30^^,^^31^^,^^32^^,^^33^^,^^34^ Although powerful, it is computationally expensive due to the extensive iterations required. Moreover, all three methods are generally limited to single-particle registration and often fail in the presence of multiple particles, underscoring a significant limitation in realistic scenarios.

In step 2 (spectral alignment), all other energy projections are registered to the baseline tomogram established in step 1. Both experimental and computational approaches attempt to address the difficulty of aligning projections across different X-ray energies. Alignment methods generally fall into three categories. The first relies on mutual information metrics, such as fast normalized cross-correlation (CC)^35^ and sum of squared differences.^36^ The second uses feature-based approaches that leverage feature detection and matching algorithms to align images. Representative methods include the scale-invariant feature transform (SIFT),^37^ speeded-up robust features,^38^ and optical flow.^39^ However, these methods often struggle with high noise, artifacts, and significant intensity variations across energy levels. The third category consists of deep-learning-based approaches, including unsupervised methods such as the spatial transformer network (STN)^40^ and supervised methods such as local feature transformer (LoFTR).^41^ Developed primarily for natural images, these deep learning methods often perform poorly on XANES data due to energy-dependent variations in brightness, contrast, and background. Although significant efforts have been made to address complex scenarios involving non-rigid sample transformations,^20^^,^^39^^,^^42^ no computationally efficient, robust, and unified data-driven framework has yet been proposed for spectro-tomographic alignment.

To overcome these limitations, we introduce CANet (coordinate-based alignment network), a data-driven approach that leverages self-supervised learning to correct deformations in X-ray spectro-tomography. CANet is built on the concept of implicit neural representations (INRs),^43^^,^^44^ in which a neural network learns a continuous mapping from input coordinates (in our case, projection angle and energy) to an output (the parameters of a geometric transformation). The geometric model is restricted to translation and scaling, as rotation and shear are assumed negligible due to the inherent imaging physics. This coordinate-based framework is especially effective for modeling the complex, continuous deformations that occur during spectro-tomographic acquisition. A key advantage of CANet is its self-supervised design: it is trained directly on the experimental data it aims to correct, requiring no external training data or fiducial markers. The network learns to predict the affine transformations that maximize the consistency of the entire dataset, simultaneously registering images across both the angular and spectral dimensions.

We demonstrate the effectiveness of CANet on both simulated datasets and real experimental datasets of lithium-ion battery cathode materials. Our results show that CANet robustly corrects complex deformations, significantly reducing artifacts and enhancing the fidelity of the reconstructed 3D chemical maps. This improved quality enables more accurate, precise analysis of the structural and chemical evolution of battery materials, providing crucial insights into their degradation mechanisms. By providing an efficient, accurate, and fully automated solution to the deformation problem, CANet has the potential to enable the broader application of high-fidelity X-ray spectro-tomography in materials and chemical sciences.

## Results

### CANet: A self-supervised learning framework for tomography and spectral alignment

Figure 1 illustrates the CANet framework, a unified approach for correcting deformations in X-ray spectro-tomography. As shown in Figure 1A, the workflow proceeds in two steps: tomography alignment (step 1) and spectral alignment (step 2). In step 1 (Figure 1B), the framework first aligns the highest-energy projection images for tomography, which then provides the reference for subsequent spectral alignment (Figure 1C). For tomography alignment (Figure 1B), the process consists of three stages. First, the raw projections are reconstructed into a low-resolution 3D representation of the sample. Second, this 3D reconstruction is reprojected to generate synthetic images for each of the original acquisition angles. Third, CANet is employed to align these reprojections (fixed references) with the original projections (moving images) to correct deformation (both horizontal [H] and vertical [V] shifts). To guide alignment, focal frequency loss (FFL)^45^ is used as the loss function to quantify alignment error and update the network parameters (methods section). The aligned projections are iteratively fed back into the CANet framework, where they are reconstructed and reprojected, enabling progressive refinement and improved registration accuracy with each cycle. Unlike traditional pairwise feature-matching methods, CANet simultaneously optimizes the affine parameters across all projection angles. The integration of FFL ensures efficient convergence, enabling accurate alignment without requiring extensive iterations. In step 2 (Figure 1C), CANet aligns projections across the energy dimension. The projection generated in step 1 (at the highest energy) serves as the fixed reference. CANet then adjusts the projections at all other energy points using affine transformations to match this reference. A hybrid loss function that combines FFL and CC loss is used to handle the intensity variations inherent in XANES data (methods section). Once aligned, the projections at each energy level are reconstructed into 3D volumes using standard algorithms, resulting in a fully registered 4D spectro-tomographic dataset.

Underlying both steps is a unified neural architecture (Figure 1D) based on INRs.^43^^,^^44^ The network takes 1D coordinates (angle or energy index) as input and outputs the affine parameters for deformation correction. The network is trained in a self-supervised, end-to-end manner by minimizing the difference between the transformed input images and the fixed references. This eliminates the need for external training datasets or manual annotations. Detailed network architecture, training settings, and ablation studies on activation functions and hyperparameters are provided in the methods section and supplemental information (Figures S1 and S2).

### Performance validation on simulated data for tomographic alignment

**Figure 2:**
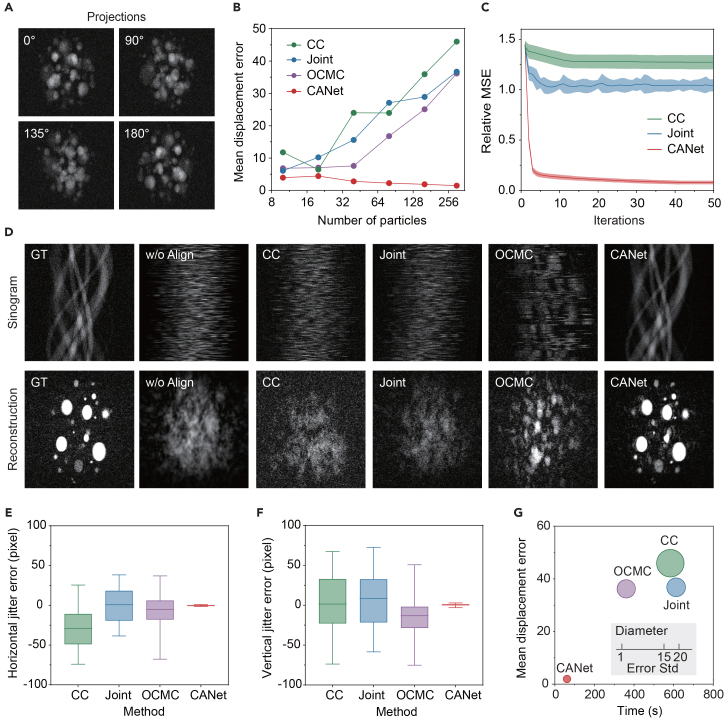
Performance evaluation on simulated data for tomographic alignment (A) Simulated projections with 300 particles in four representative views. (B) Mean displacement errors for different methods vs. the number of particles (100–300). (C) Convergence comparison of CC, Joint, and CANet using relative mean-squared error (MSE). (D) Representative sinograms and reconstructions for ground truth (GT), without (w/o) align, CC, Joint, OCMC, and CANet. (E and F) Box-and-whisker plot of horizontal and vertical jitter errors after alignment by different methods. (G) Bubble chart comparing mean displacement error and running time for different alignment methods.

We evaluated CANet’s tomographic performance on simulated data generated with the TomoPhantom framework.^46^ The analytical phantom consisted of multiple spherical objects with varying radii (Figure 2A) observed from four angles (0°, 90°, 135°, and 180°), providing 2D projections from different viewpoints. To mimic real experimental settings, Gaussian noise was introduced (details are given in the methods section). CANet’s performance was compared against established baselines: CC,^47^ Joint,^29^ and outer contour-based misalignment correction (OCMC).^18^ To ensure consistency, all reconstructions employed the compute unified device architecture (CUDA)-accelerated Gridrec algorithm implemented in Tomocupy.^48^

Alignment accuracy was quantified using the mean displacement error relative to ground truth. As shown in Figure 2B, CANet consistently achieved the lowest errors across different particle counts (10–300), demonstrating superior robustness. For instance, with 80 particles, errors for OCMC, CC, and Joint were approximately 8×, 13×, and 13× higher than those for CANet, respectively. CANet maintained high reconstruction quality across all particle counts (Figure S3) and noise levels (up to 40%, Figure S4), effectively recovering individual spheres where unaligned data failed to recover them. In the representative case (300 particles, 10% noise; Figure 2D), CANet effectively corrected edge distortions in jittered sinograms, producing clear reconstructions, whereas OCMC generated blurred results, and other methods failed to resolve smaller features. Although Joint alignment improves with SIRT reconstruction, it comes at a substantially higher computational cost. Furthermore, the Fourier shell correlation (FSC) is calculated between the projections and their corresponding reprojections from the resulting volume. FSC analysis (Figure S5A) shows that CANet extends the resolvable frequency to 0.35 pixel^−1^ (at FSC = 0.5), significantly outperforming the unaligned baseline, which drops sharply at low frequencies.

Figure 2C compares the convergence of iterative methods (CC, Joint, and CANet) using relative mean-square error (RMSE) as a criterion (OCMC excluded as non-iterative). CANet demonstrated significantly faster convergence, stabilizing within 10 iterations across all noise levels and particle counts (Figures S6A and S6B). Consequently, 10 iterations were used in all experiments to balance accuracy and efficiency. Quantitative comparisons (Figures 2E and 2F) show that CANet achieved the lowest H and V jitter errors (1.32 and 2.67 pixels, respectively), which are approximately 20× lower than those of other methods. Figure 2G further highlights CANet’s superior balance of accuracy and efficiency: it reduces the mean displacement error by 20×–25×, the runtime by 3×–5×, and the error standard deviation by 20×–25× compared to CC, Joint, and OCMC.

### Performance validation on simulated data for spectral alignment

**Figure 3:**
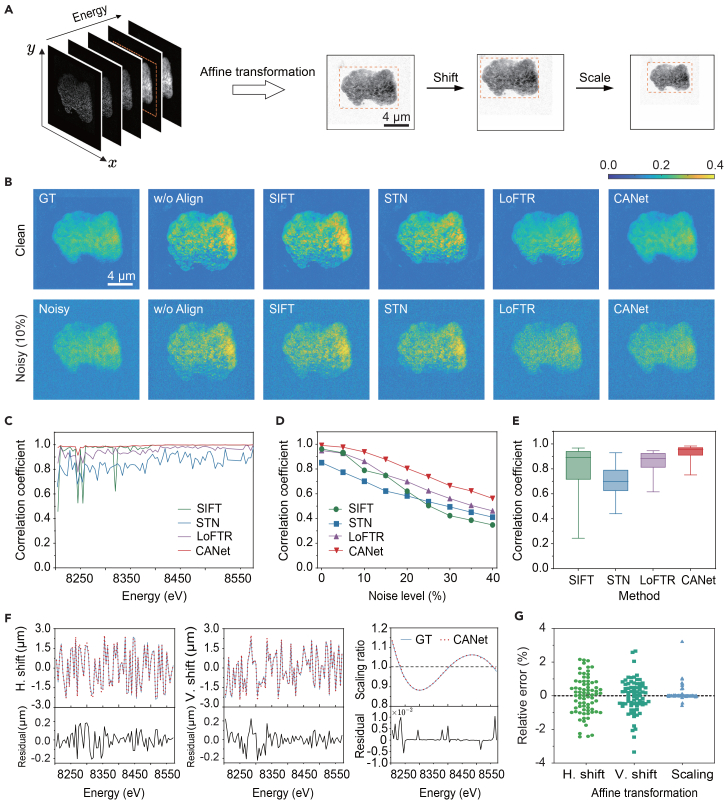
Performance evaluation on simulated data for spectral alignment (A) Synthetic XANES data containing affine transformations (shift and scaling). (B) Absolute residual error maps comparing alignment results. (C) Pearson correlation coefficient across different methods and energy levels. (D) Robustness under varying noise levels, measured by the average Pearson correlation coefficient. (E) Pearson correlation coefficients distribution for aligned data with 10% noise. (F) Affine transformation parameters estimated by CANet compared to GT. (G) Relative error of affine transformation parameters under noise-free conditions.

We evaluated CANet’s spectral alignment performance on a synthetic XANES image stack generated using TomoBank.^49^ The dataset contained 75 energy-dependent projections of a single tomogram, with applied affine transformations (shift and scale) and Gaussian noise (Figure 3A). Baselines included SIFT^37^ and the deep learning approaches STN^40^ and LoFTR.^41^

Visual assessment using residual maps (Figures 3B and S7A) revealed that baseline methods struggled with the intensity and contrast variations inherent to XANES spectra, resulting in evident artifacts and edge distortions. In contrast, CANet consistently maintained fewer residual artifacts across all noise levels, resulting in cleaner results. Quantitatively, CANet demonstrated superior alignment fidelity, achieving a mean Pearson correlation coefficient of 0.991, significantly outperforming SIFT (0.962), LoFTR (0.950), and STN (0.851) (Figure 3C). Figure 3D demonstrates the robustness of CANet against noise. Specifically, although feature-based SIFT worked well under low noise, its accuracy dropped significantly as the noise level increased. In contrast, CANet outperformed all other methods even under an extreme noisy condition (40% noise). Under lower noise levels, for instance, at 10% noise, CANet (0.94 ± 0.04) performed much better than SIFT (0.79 ± 0.21), LoFTR (0.86 ± 0.07), and STN (0.70 ± 0.11) (Figures 3E and S7B).

A fine-grained comparison between the ground truth and CANet-estimated transformation parameters (H and V shifts, scaling ratios) is shown in Figure 3F. The maximum residual jitters for H and V directions were below 0.2 μm, indicating high alignment precision. Relative alignment errors were computed as discrepancies between estimated and ground-truth values normalized by their respective magnitudes. As summarized in Figures 3G and S8, under noise-free conditions, CANet achieved mean relative errors below 1% (H shift: 0.87%; V shift: 0.75%; scale: 0.15%), with maximum errors around 3% (H shift: 2.16%; V shift: 2.65%; scale: 3.23%). Even under moderate- and high-noise conditions, its mean relative errors remained below 5% (≤25% noise), confirming CANet’s robustness.

### Application to nano-tomography and spectro-tomography of battery cathode particles

To demonstrate generalizability, we applied CANet to two complementary experimental datasets of cathode particles: an NMC622 (LiNi_0.6_Mn_0.2_Co_0.2_O_2_) nano-tomography dataset for tomographic alignment and a distinct LCO (LiCoO_2_) spectro-tomography dataset for spectral alignment (see methods for details).

**Figure 4:**
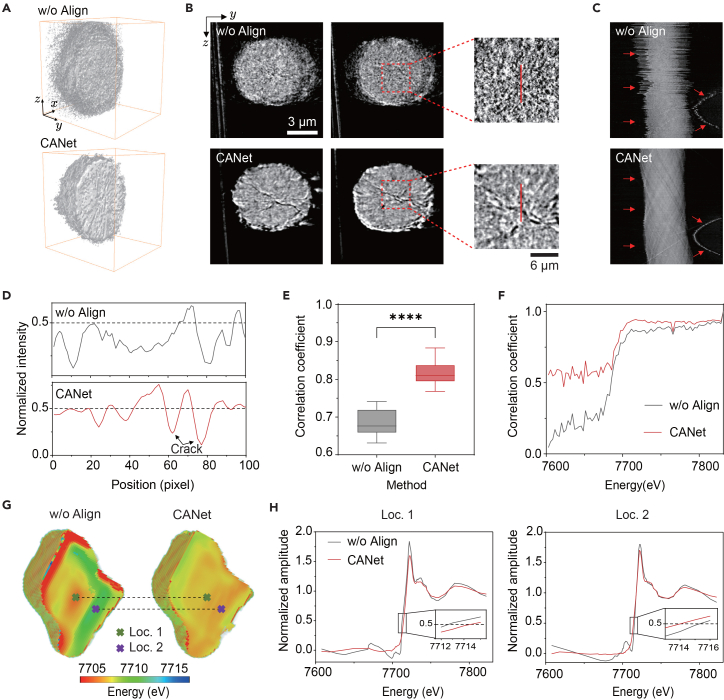
Real-world application of CANet for aligning nano-tomographic and spectro-tomographic projections of battery cathode particles (A) 3D reconstructions comparing unaligned and CANet-aligned projections. (B) Representative slices with zoomed-in views of regions of interest. (C) Sinograms from unaligned and CANet-aligned projections. (D) Intensity profiles along the highlighted crack lines in (B). (E) Comparison of mean correlation coefficients across projections at different energies between unaligned and CANet-aligned data (*n* = 180, ∗∗∗∗*p* < 0.0001). (F) Correlation coefficients across energies for a representative projection. (G) 3D chemical maps reconstructed with unaligned and CANet-aligned data. (H) XANES spectra corresponding to the selected pixels in (G) (locations 1 and 2).

CANet significantly enhanced the reconstruction quality of the NMC622 particle. As shown in Figures 4A–4C, the aligned 3D volume and 2D slices reveal a clearer visualization of cracks, effectively suppressing the non-uniform motion artifacts and blurring observed in unaligned data. Furthermore, CANet outperforms baselines (Figure S9), particularly in resolving peripheral details and internal cracks. Unlike the contour-based OCMC, which suffers from blurred interfaces and center-of-rotation (CoR) misalignments, CANet reconstructs sharp features with high geometric fidelity. Quantitative intensity profiles (Figure 4D) further confirmed the precise localization of cracks, which is critical for understanding chemomechanical degradation.^50^ CANet demonstrated remarkable efficiency and robustness, achieving convergence within 10 iterations (<2 min) (Figures S10C and S10D) while significantly enhancing reprojection quality and signal-to-noise ratio (SNR) compared to unaligned data (Figure S10B).

Spectral alignment is further evaluated on the spectro-tomography dataset. As shown in Figure 4E, CANet improved the average correlation coefficient by 0.14 (0.68 ± 0.03 for unaligned data vs. 0.82 ± 0.02 for CANet-aligned data) across projections at all energy points. As shown in Figure 4F, CANet alignment yielded consistently higher correlation coefficients (0.79 ± 0.17) compared to the unaligned data (0.63 ± 0.32) for the representative projection. The reconstructed 3D chemical map (Figure 4G) shows that CANet substantially enhances contrast and resolution compared with the unaligned reconstruction, enabling clearer chemical and structural interpretation. Corresponding 2D slices (Figure S11B) confirm a smoother spatial distribution at the 0.5 edge-point energy. XANES spectra from selected pixels (Figure 4H) reveal an observable edge-point shift following correction, which originates from residual jitter exceeding 30 pixels in the unaligned data (Figure S11C). Given that the edge-point energy (at 0.5 normalized intensity) is a critical proxy for chemical-state transitions,^51^^,^^52^ CANet’s ability to eliminate geometric misalignment is vital. It enables the precise quantification of oxidation states, ensuring reliable interpretation of the cathode’s chemomechanical behavior.

### Application to a realistic spectro-tomography of a heterogeneous NMC battery cathode particle

We further validated CANet on a benchmark spectro-tomography dataset (heterogeneous NMC) from TomoBank,^49^ acquired via nano-computed tomography (nano-CT) with a resolution of 33.4 nm. This dataset presents a “blind” challenge with unknown joint offsets in both tomographic and spectral dimensions.

**Figure 5:**
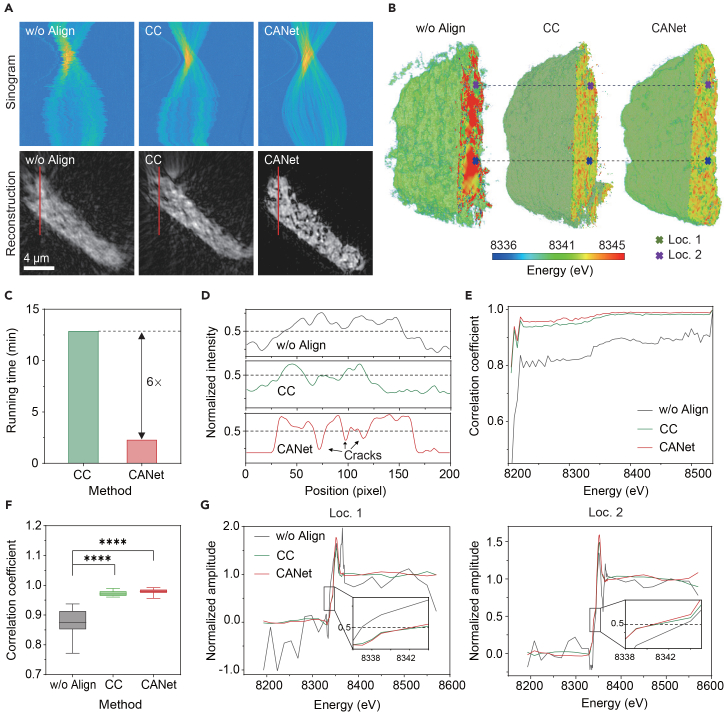
Real-world application of CANet to spectro-tomography of a heterogeneous NMC battery cathode particle (A) Representative sinograms and reconstructed slices of projections comparing unaligned, CC, and CANet results. (B) 3D chemical mapping of the particle for each method. (C) Computational runtime comparison between CC and CANet. (D) Intensity profiles along the highlighted crack region in (A). (E) Correlation coefficients across different energies for a representative tomogram. (F) Distribution of mean correlation coefficients across all projections (*n* = 180,∗∗∗∗*p* < 0.0001). (G) XANES spectra from locations 1 and 2 marked in (B).

For tomographic alignment, Figures 5A and S12B visualize unaligned and CC- and CANet-aligned results. CANet achieved significantly better alignment results, while both the unaligned and CC-aligned results exhibited coarser structural details. Furthermore, CANet notably outperforms baseline methods (Figure S13), particularly in resolving internal particle cracks within the reconstruction slices. Whereas OCMC struggles to maintain clarity, CANet delivers markedly sharper definitions of both cracks and finer structural features. Intensity profiles (Figure 5D) demonstrate that CANet reveals sharper crack boundaries and finer features with smoother transitions than the other methods. Furthermore, CANet achieved this with superior computational efficiency, completing the alignment significantly faster than the CC method (Figure 5C).

For spectral alignment, CANet consistently outperformed baselines. The overall correlation coefficient across projections at all energy points improved to 0.98 ± 0.01 for CANet, compared to 0.97 ± 0.01 for CC and 0.88 ± 0.03 for unaligned data (Figure 5F). The 3D chemical map (Figure 5B) with CANet-aligned data showed fewer edge artifacts than the CC-aligned map, which suffered from distortions visible in the *xz*/*yz* slices (Figure S14A).

The extracted XANES spectra (Figure 5G) demonstrate that both CC and CANet corrected the edge-point shift relative to the unaligned data. Additionally, CANet’s estimated jitter profiles (Figure S14B) revealed a subtle, energy-dependent scaling drift likely caused by focal changes in the X-ray optics, a distortion that the CC method is unable to correct. These results confirm that CANet provides a robust, efficient, and high-fidelity solution for complex spectro-tomographic reconstruction, proving a valuable tool for characterizing complex battery chemomechanics.

## Discussion

The alignment of X-ray spectro-tomography projections is a fundamental prerequisite for reliable nanoscale chemical imaging. Mechanical instabilities and thermal drift during the prolonged acquisition times required for 4D datasets introduce motion artifacts that degrade spatial resolution and distort spectral fidelity. These errors compromise the ability to correlate local morphology with chemical state, creating a bottleneck in fields ranging from energy storage to catalysis.

In this study, we introduced CANet, a self-supervised coordinate-based network that unifies tomographic and spectral alignment. Unlike supervised deep learning methods, CANet does not rely on ground truth or require external training data, enabling direct application to experimental datasets without pretraining. For tomographic alignment, CANet demonstrated exceptional robustness, reducing displacement errors to subpixel levels (0–3 pixels) even under high-noise conditions, whereas conventional methods such as OCMC and Joint alignment failed. Crucially, it can handle multiple particles in a large field of view, where center-of-mass or contour-based algorithms typically fail, thereby enabling its application to real battery imaging data.

When applied to battery materials, CANet enabled the visualization of fine intragranular cracks and the accurate mapping of oxidation states. In spectral alignment, CANet’s ability to handle the non-linear intensity variations of XANES edges allowed it to outperform feature-based methods (SIFT) and standard deep learning approaches (LoFTR). By correcting energy-dependent magnification and shifts, CANet ensured that the spectral features extracted from the 3D volume precisely reflected the material’s local chemistry rather than motion artifacts.

CANet currently models deformations using affine transformations (translation and scaling). While this addresses most instrumental instabilities, it does not capture non-rigid deformations that can arise in soft materials or during *operando* experiments with large volume changes. Future work will focus on extending the CANet framework to handle non-rigid deformations and integrating physics-informed constraints to better represent complex material behaviors. Additionally, we plan to integrate CANet into real-time data-processing pipelines at synchrotron facilities, leveraging its computational efficiency to provide on-the-fly experimental feedback.

## Methods

### Proposed alignment method

Image alignment aims to determine the optimal affine transformation such that the target image aligns with the source image. Mathematically, we consider two images T(x),S(x):x∈Ω→R with a bounded domain $\Omega \subset R^{2}$; our objective is to minimize an optimization problem expressed as

(Equation 1) $$\min_{v}L\left(S\left(x\right),T\circ \phi_{v}\left(x\right)\right)\text{,}$$

where the loss function *L*(·,·) quantifies the similarity between these two images and ∘ is the composition of the function. Given that rotation and shear are negligible due to the inherent imaging physics, we restrict the geometric model to translation and scaling. For simplicity, without loss of generality, we reduce the transformation matrix to a 2 × 3 matrix by removing the last row from Figure 1, resulting in

$$\phi_{v}\left(x\right)=\left[\begin{array}{ccc} s_{x} & 0 & t_{x}\\ 0 & s_{y} & t_{y} \end{array}\right]\left[\begin{array}{l} x\\ 1 \end{array}\right]\text{,}$$

where the affine vector $v:={\left[s_{x},s_{y},t_{x},t_{y}\right]}^{T}\in R^{4}$ contains the scaling factors ($s_{x},s_{y}\in R$) along the *x* and *y* directions and the corresponding displacement factors ($t_{x},t_{y}\in R$). In the tomographic alignment task, we only consider the displacement case where $\phi_{v}\left(x\right)=x+{\left[t_{x},t_{y}\right]}^{T}$ with $v={\left[t_{x},t_{y}\right]}^{T}$. The projections are structured as a stack of *n* 2D images, each of size h × w. The interpretation of the third dimension is modality dependent: it corresponds to either an angle-specific projection (tomography) or an energy-specific projection (spectral).

For tomographic alignment, we utilize the projection-reprojection approach to iteratively update the displacement. In each iteration, we have a series of 2D projection images at different angles $S\in R^{h\times w\times n}$ as the source images. Using this reconstructed 3D volume, we then generate the reprojections at the same angles: T=g∘f(S), where *f* and *g* are the reconstruction and projection operators, respectively. If T is not similar to S, then there is a tomographic misalignment. To align the projections at different angles, we align each front slice between these two tensors via the following optimization problem:

(Equation 2) $$\min_{V}\sum_{i=1}^{n}L\left(S^{\left(i\right)}\left(x\right),T^{\left(i\right)}\circ \phi_{v^{\left(i\right)}}\left(x\right)\right),s.t.\;T=g\circ f\left(S\right),V=\left[v^{\left(1\right)},v^{\left(2\right)},\ldots ,v^{\left(n\right)}\right]\text{.}$$

For spectral alignment, we consider a series of 2D projections acquired at different energy points, $S=\left[S^{\left(1\right)},S^{\left(2\right)},\ldots ,S^{\left(n\right)}\right]\in R^{h\times w\times n}$. Here, the source image is the front slice with the highest energy. Without loss of generality, we permute this slice as the last one. The remaining projections **S**^(*i*)^ for *i* = 1, …,*n* − 1 serve as the target images to be aligned. Consequently, the alignment optimization problem is formulated as

(Equation 3) $$\min_{V}\sum_{i=1}^{n-1}L\left(S^{\left(n\right)}\left(x\right),S^{\left(i\right)}\circ \phi_{v^{\left(i\right)}}\left(x\right)\right),s.t.\;V=\left[v^{\left(1\right)},v^{\left(2\right)},\ldots ,v^{\left(n-1\right)}\right]\text{.}$$

We propose a unified framework based on INRs.^43^^,^^44^ Instead of solving for discrete alignment parameters, we parameterize the affine vector **v**^(*i*)^ as a continuous function of the normalized coordinate *c*_*i*_∈[−1,1] (representing angle or energy). This mapping is learned by a neural network Ψ_Θ_: **v**^(*i*)^ = Ψ_Θ_(*c*_*i*_), where Θ denotes the network parameters. The architecture consists of a multilayer perceptron (MLP) utilizing a periodic sine activation function.^44^ Specifically, the *j*-th layer is defined as

(Equation 4) $$\psi_{j}\left(c_{i}\right)=\sin\left(\omega_{j}*W_{j}\psi_{j-1}\left(c_{i}\right)+b_{j}\right)\text{,}$$

where *ω*_*j*_ is the frequency of the *j*-th layer and **W**_*j*_ and **b**_*j*_ are the weight and bias, respectively. Here, *ψ*_*j*−1_ is the previous layer’s output (or the input coordinates for *j* = 1). Here, we can reformulate the tomographic and spectral alignment optimization problems (Equations 2 and 3) as

(Equation 5) $$\min_{\Theta_{t}}\sum_{i=1}^{n}L\left(S^{\left(i\right)}\left(x\right),T^{\left(i\right)}\circ \phi_{v^{\left(i\right)}}\left(x\right)\right),s.t.\;T=g\circ f\left(S\right),v^{\left(i\right)}=\Psi_{\Theta_{t}}\left(c_{i}\right),\text{for}\;\text{tomographic}\;\text{alignment},\text{and}$$

$\min_{\Theta_{s}}\sum_{i=1}^{n-1}L\left(S^{\left(n\right)}\left(x\right),S^{\left(i\right)}\circ \phi_{v^{\left(i\right)}}\left(x\right)\right),s.t.\;v^{\left(i\right)}=\Psi_{\Theta_{s}}\left(c_{i}\right)\text{,}$ for spectral alignment.

Furthermore, these networks provide a more compact representation of a continuous function and facilitate smooth manipulation. Note that the input to the MLP is the coordinate of projection coordinates (i.e., angle or energy indices), which are independent of the spatial resolution of the XANES dataset. Consequently, this architecture enables the network to perform registration by jointly aligning the entire image stack.

### Loss function in tomography and spectral alignment

The loss function differs between tomographic and spectral alignment, based on the specific requirements of these tasks: for tomographic alignment, we utilize the FFL^45^ to optimize the alignment. The loss function *L*(·,·) in Equation 5 is formulated as

(Equation 6) $$L_{\text{tomo}}\left(S,T\right)=\frac{1}{\left|\Omega \right|}\sum_{k\in \Omega }w\left(k\right){\left|\bar{S}\left(k\right)-\bar{T}\left(k\right)\right|}^{2}\text{,}$$

where $w\left(k\right)={\left|\bar{S}\left(k\right)-\bar{T}\left(k\right)\right|}^{\alpha }$ is the spectrum weight matrix with a scale parameter *α* for flexibility and $\bar{S}$ and $\bar{T}$ represent the discrete Fourier transform (DFT) of two images, respectively. Here, |Ω| is the discrete volume of Ω, i.e., |Ω| = *h*∗*w*. To ensure consistent alignment, the tomographic loss is averaged over every projection angle. This loss ensures the moving reprojection matches the fixed projection at every angle in the frequency domain, which is critical for achieving an accurate alignment of projections.

For spectral alignment, the loss function combines the FFL and the correlation-based term to account for both frequency-domain and spatial-domain differences. The loss function *L*(·,·) in Equation 5 is defined as

(Equation 7) $$L_{\text{spec}}\left(S,T\right)=\frac{1}{\left|\Omega \right|}\sum_{k\in \Omega }w\left(k\right){\left|\bar{S}\left(k\right)-\bar{T}\left(k\right)\right|}^{2}-\lambda \frac{\left\langle S,T\right\rangle }{∥S∥∥T∥}\text{,}$$

where ⟨·,·⟩ denotes the inner product and ∥·∥ is the Euclidean norm of the matrix. The parameter *λ* balances the contributions of the spatial-domain and frequency-domain losses. The loss is computed for each energy level by comparing the reference **S**^(*n*)^ with the corresponding moving image **S**^(*i*)^ at the *i*-th energy and then averaged across all energy levels to ensure consistent alignment over the energy spectrum.

### Implementation details

#### Network architecture and training details

The CANet architecture consists of a 3-layer MLP with 256 hidden units and a frequency parameter *ω*_0_ = 30 per layer. Details regarding the selection of the activation function are provided in Figure S2. Implementation was performed in PyTorch 1.21.1 on an NVIDIA L40 GPU. CANet’s parameters are optimized with the Adam optimizer using an initial learning rate of 1 × 10^−4^, with the learning rate decay controlled by a ReduceLROnPlateau scheduler (patience: 10, decay: 0.9). The CANet is trained for 200 epochs, with the tomographic projection-reprojection procedure iterated 10 times.

#### Data preprocessing

Raw projections were first flat-field corrected and converted to absorbance using the Beer-Lambert law (*A* = −*ln*(*I*/*I*_0_)), followed by image cropping. To mitigate angle-dependent intensity variations caused by the capillary holder, background subtraction was performed prior to the registration workflow. Finally, 3D reconstruction and XANES analysis were conducted using TXM-Wizard.^53^

#### Performance evaluation metrics

To quantitatively evaluate the performance of CANet, we utilized both synthetic and experimental datasets. For synthetic tomographic alignment, where ground truth is available, the H (Δ**x**) and V (Δ**y**) jitter errors represent the displacement vectors between the aligned projections and ground truth for all angles along the *x* and *y* directions, respectively. The displacement error is defined as the Euclidean norm of the displacement vectors:

(Equation 8) $$\varepsilon =\sqrt{\Delta x^{2}+\Delta y^{2}}\text{.}$$

This formulation quantifies the misalignment, with displacement error providing a measure of the error in pixel or subpixel units. The mean displacement error is computed as the average of the displacement error vector over all angles. For experimental data, Δ**x** and Δ**y** represent the magnitude of jitter output by CANet and CC in each iteration along the *x* and *y* axes, respectively. The displacement error was calculated as described in Equation 8.

In spectral alignment, quantitative evaluation on synthetic data was performed using the Pearson correlation coefficient (*δ*) between the aligned projection and ground truth:

(Equation 9) $$\delta =\frac{\sum_{i=1}^{n}\left(x_{i}-\bar{x}\right)\left(y_{i}-\bar{y}\right)}{\sqrt{\sum_{i=1}^{n}{\left(x_{i}-\bar{x}\right)}^{2}}\sqrt{\sum_{i=1}^{n}{\left(y_{i}-\bar{y}\right)}^{2}}}\text{,}$$

where *x*_*i*_ and *y*_*i*_ are the intensities of the *i*-th pixel in the aligned and ground-truth images, respectively, with $\bar{x}$ and $\bar{y}$ representing their mean values. For experimental datasets, the correlation was computed relative to the highest-energy reference projection to assess alignment consistency.

#### Details of simulation experiments

To evaluate performance under controlled conditions, we generated two synthetic datasets. (1) Tomographic alignment: an analytical phantom was created using the TomoPhantom,^46^ comprising 402 projections (256 × 362 pixels) over 0°–180°. We introduced random H and V jitters (±75 pixels) and varying levels of Gaussian noise (10%, 20%, 30%, and 40% of the maximum signal intensity). (2) Spectral alignment: a synthetic XANES image stack was generated from a real cathode tomogram (TomoBank: 00089^49^). Using a representative projection at 60°, we generated 75 energy-dependent images (1,024 × 1,024 pixels). The alignment was done up to an image size of 331 × 402 pixels. Deformations included affine shifts (±3 μm), continuous scaling, and Gaussian noise (5%–40%) to test robustness.

#### Details of real experimental data

We validated CANet using three experimental datasets acquired at different synchrotron facilities. Dataset 1: NMC622 (tomography), for which nano-tomographic data were collected at beamline 4W1A of the Beijing Synchrotron Radiation Facility (BSRF). A total of 180 projections were recorded at 8,359 eV over 180°. The raw images (1,024 × 1,024 pixels, 27 nm resolution) were cropped to 400 × 400 pixels to eliminate peripheral illumination artifacts. Dataset 2: LCO (spectro-tomography), for which data were acquired at the Shanghai Synchrotron Radiation Facility (SSRF). The dataset spans 83 energy points (7,608–7,728 eV) across the transition metal edge. Each energy level comprises 180 projections over 180°. Raw images (240 × 308 pixels, 58 nm resolution) were cropped to 150 × 230 pixels for processing. Dataset 3: heterogeneous NMC (spectro-tomography), for which nano-CT data were collected at the Stanford Synchrotron Radiation Lightsource (SSRL). This dataset includes 67 energy points (6,534–8,570 eV), specifically selected to capture the K-edges of Mn, Co, and Ni for elemental mapping, as well as the Ni K-edge XANES for valence-state analysis. Each energy consists of 180 projections over 180°. The raw images (1,024 × 1,024 pixels, 33 nm resolution) were cropped to an effective field of view of 331 × 402 pixels to minimize edge artifacts.

### Ablation studies

#### Sensitivity analysis of loss functions and hyperparameters

Ablation studies on loss functions and hyperparameter selection are detailed in Figure S1. Based on these simulation results, we maintained consistent hyperparameters across all experimental datasets to ensure reproducibility. Specifically, the FFL scale parameter was set to *α* = 1 for both alignment tasks, while the spectral balancing parameter was fixed at *λ* = 0.1.

#### Quantitative resolution estimation via FSC

FSC was employed to quantitatively assess the reconstruction quality. The correlation is calculated between the projections and their corresponding reprojections generated from the 3D reconstruction. As illustrated in Figure S5, the nominal resolution was determined using the FSC = 0.5 criterion. For the simulation, NMC622, and heterogeneous NMC datasets, the CANet-aligned data maintain significantly higher correlation at high frequencies compared to the unaligned baseline, where correlation drops rapidly even at low frequencies. These results confirm that CANet effectively restores high-resolution structural details across simulation and real spectro-tomographic datasets.

#### Application of CANet to CoR correction

An important and common source of artifacts in tomographic reconstruction is the offset of the CoR and tilt of the rotation axis. To mitigate reconstruction artifacts induced by CoR misalignments, we extended the CANet framework to CoR correction. In numerical simulations, we introduced random H CoR offsets of ±5 and ±10 pixels to the standard CoR, with the simulation projections shown in Figure 2. As illustrated in Figure S15, while CoR offsets lead to significant structural artifacts, CANet effectively compensates for these shifts and eliminates the artifacts. Reconstructed slices were quantitatively evaluated using peak SNR (PSNR) relative to the standard CoR (0 pixel) baseline. CANet achieves substantially higher PSNR values than uncorrected slices.

#### Sensitivity to initial jitter magnitude

We conducted a sensitivity analysis to test the effect of initial jitters on alignment accuracy, as larger initial jitters lead to lower resolution in the initial reconstructed volume. As illustrated in Figure S16A, the displacement errors for unaligned and CANet vary with the initial jitter magnitude. We observe that CANet consistently improves the reconstruction quality, even when the initial jitter magnitudes are increased. Additionally, we present some representative sinograms and reconstruction slices in Figures S16B and S16C, respectively.

#### Robustness against spectral contrast variations

For spectral alignment, to address substantial intensity fluctuations near absorption edges, we formulated a composite loss function that combines the FFL and the cross-hybrid loss based on structural dominance over absolute intensity. Specifically, the alignment relies primarily on high-frequency spatial features (such as edges and boundaries) rather than absolute intensity values. These structural features are reflected in the relative intensity within each energy level and remain stable even when the energy varies. We conducted further experiments to evaluate the CANet performance between projections exhibiting strong spectral contrast variations (e.g., 8,253 vs. 8,348 eV). The visualizations of the projections and the absolute residual maps after alignment are shown in Figures S17A and S17B. CANet maintains robustness and accuracy even under significant spectral contrast changes. Additionally, quantitative assessment (Figure S17C) demonstrates that the CC coefficient with the reference (8,570 eV) remains high, confirming the method’s robustness to strong spectral contrast variations.

#### Comparison of grid interpolation methods

The spatial transformation (Warp) is implemented using a differentiable grid sampling operation. Since the estimated affine parameters yield continuous (non-integer) coordinates, we utilize bicubic interpolation to sample pixel intensities at subpixel locations, with zero padding applied to out-of-bound coordinates. We evaluated both bilinear and bicubic interpolation for resampling. As shown in Figure S18, bicubic interpolation yields superior performance. Specifically, the reconstructed slices exhibit significantly sharper crack features with bicubic interpolation than with bilinear interpolation. In addition, CANet operates in an iterative mode, allowing artifacts from interpolation in early stages to be eliminated during subsequent iterations. This has been evaluated by the reduction of errors among training iterations in Figure S18.

## Resource availability

### Lead contact

Requests for further information and resources should be directed to the lead contact, Jizhou Li (jzli@ee.cuhk.edu.hk).

### Materials availability

This study did not generate new materials.

### Data and code availability

The source code to create the figures and perform the analysis is available at GitHub (https://github.com/wangting1907/CANet) and has been archived at Zenodo^54^ (https://doi.org/10.5281/zenodo.18512598).

## Acknowledgments

This work was partially supported by the 10.13039/501100001809National Natural Science Foundation of China (grant nos. T2422017, 52303301, 12571564, and 52325207), the Guangdong Basic and Applied Research Foundation (2024A1515012347), the Hong Kong Research Grants Council (21204124), the 10.13039/501100006355Shun Hing Institute of Advanced Engineering (RNE-p1-25), and a 10.13039/501100004853CUHK direct grant (4055248).

## Author contributions

J.L., C.W., and X.Y. conceived and supervised the project. T.W. and Z.Y. implemented the alignment model with the guidance of M.K.-P.N., C.W., and J.L. Experimental data collection was carried out by H.P. and K.Z. T.W., Z.Y., C.W., and J.L. wrote the manuscript with input from all authors. All authors discussed the results and contributed to the final version of the paper.

## Declaration of interests

The authors declare no competing interests.

## Supplemental information

Document S1. Figures S1–S18

Document S2. Article plus supplemental information
