# Supplementary material for: Data-driven deformation correction in X-ray spectro-tomography with implicit neural networks
Source: Patterns (N Y). 2026 Mar 30;7(5):101515. doi: 10.1016/j.patter.2026.101515 (PMC13161694; doi:10.1016/j.patter.2026.101515)
Supplement: Document S1. Figures S1–S18 [file mmc1.pdf]

Patterns, Volume *7*

Supplemental information

Data-driven deformation correction in X-ray spectro-tomography with implicit neural networks

Ting Wang, Zipei Yan, Hongyi Pan, Kai Zhang, Michael K.-P. Ng, Xiqian Yu, Chao Wang, and Jizhou Li

Figure S1. Evaluation of loss functions and hyperparameter optimization.

Figure S2. Evaluation of activation functions.

Figure S3. Tomographic alignment performance with different particle counts.

Figure S4. Tomographic alignment performance under varying noise levels.

Figure S5. Fourier shell correlation analysis.

Figure S6. Trade-off study between convergence efficiency and computational cost.

Figure S7. Spectral alignment performance under varying noise levels.

Figure S8. Affine parameter estimations under noisy conditions for spectral alignment.

Figure S9. Alignment performance of different methods for NMC622.

Figure S10. Tomographic preprocessing and alignment for NMC622.

Figure S11. Spectral preprocessing and alignment for LCO.

Figure S12. Tomographic preprocessing and alignment for Heterogeneous NMC.

Figure S13. Alignment performance of different methods for Heterogeneous NMC.

Figure S14. Spectral preprocessing and alignment for Heterogeneous NMC.

Figure S15. Robustness against center of rotation offsets.

Figure S16. Impact of jitter magnitude on alignment accuracy.

Figure S17. Energy-dependent correlation analysis.

Figure S18. Visual comparison between bilinear and bicubic interpolation methods.

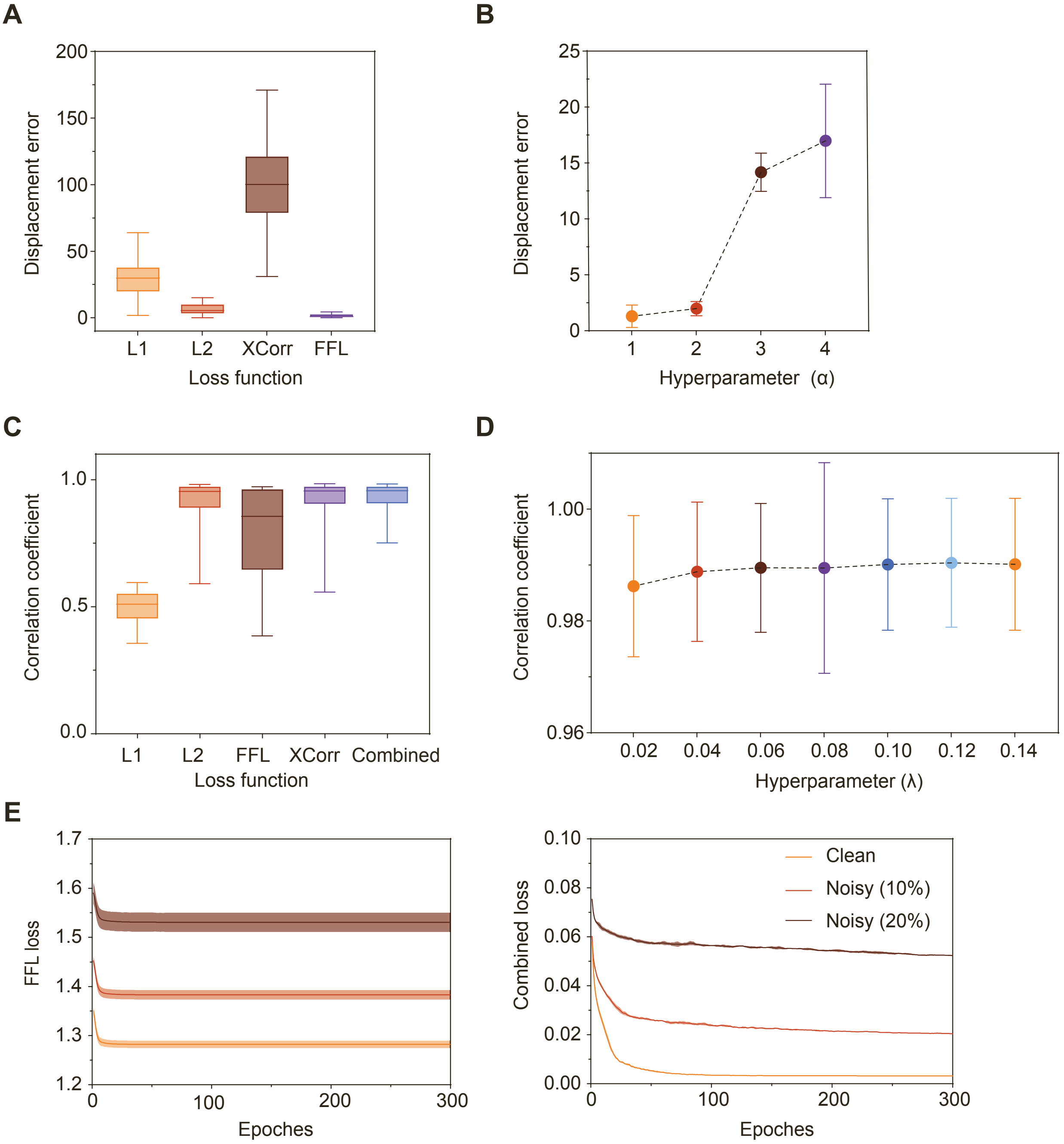

Figure S1. **Evaluation of loss functions and hyperparameter optimization.** (A) Four loss functions are considered for tomographic alignment and quantified by the displacement error, where the FFL outperformed others. (B) Hyperparameter α study of the FFL for tomographic alignment, where $\alpha = 1$ demonstrated better performance. (C) Evaluation of five loss functions for spectral alignment, where the hybrid loss achieves the highest correlation. (D) Ablation study of λ of the hybrid loss function, where $\lambda = 0.12$ outperformed others. (E) Visualizes the training convergence for these optimal settings under both clean and noisy (10%, 20%) conditions.

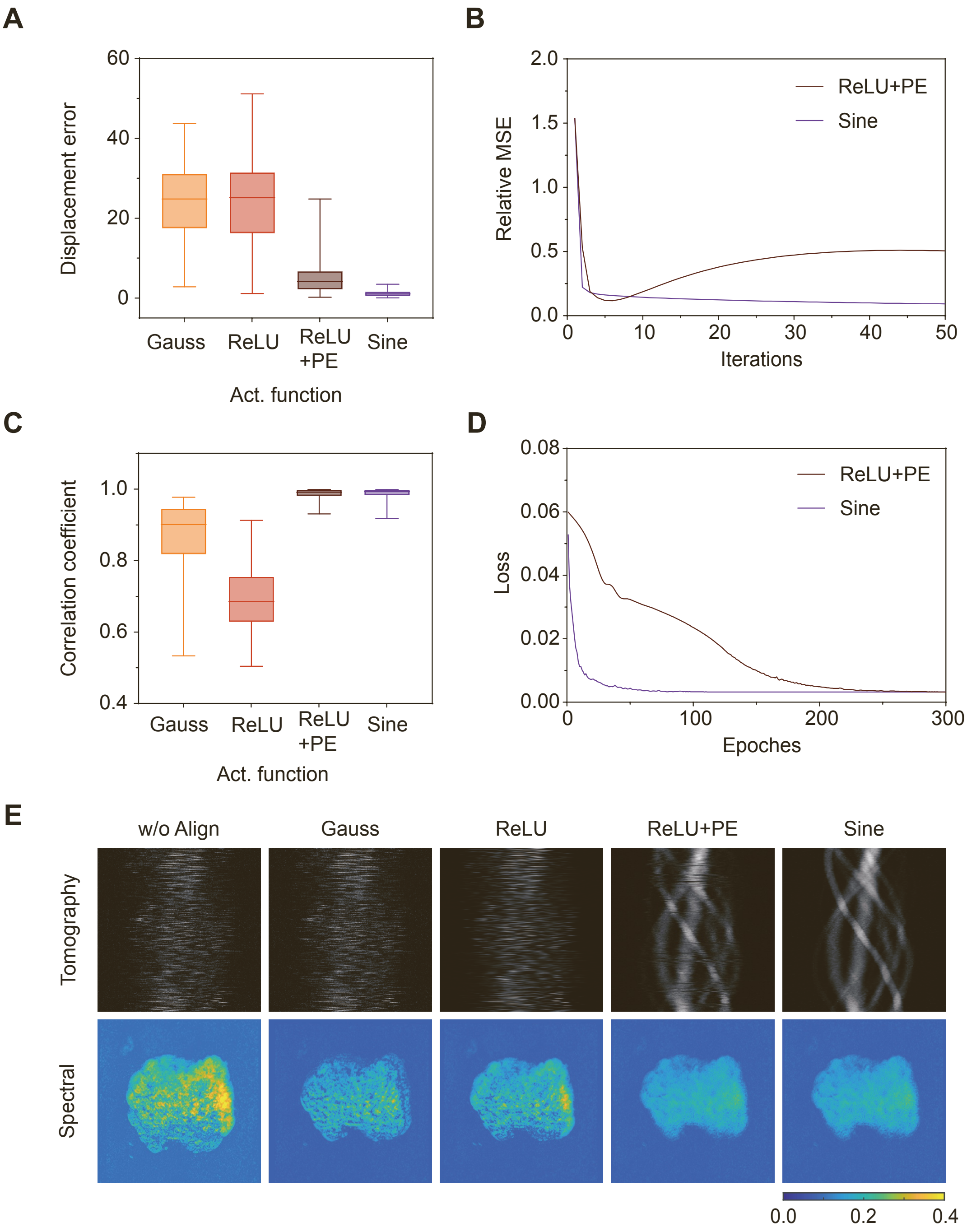

Figure S2. **Evaluation of activation functions.** (A) Comparison of displacement errors for tomographic alignment across four activation functions; Sine yield the lowest errors. (B) Relative MSE curves over projection-reprojection iterations, showing that Sine exhibits stable convergence, whereas ReLU+PE suffers from overfitting in later stages. (C) Performance comparison for spectral alignment, where Sine and ReLU+PE again outperform others. (D) Loss curves over 300 epochs, demonstrating the faster convergence of Sine. (E) Qualitative assessment via reconstructed sinograms (tomographic) and absolute residual maps (spectral), visually confirming the superiority of the Sine.

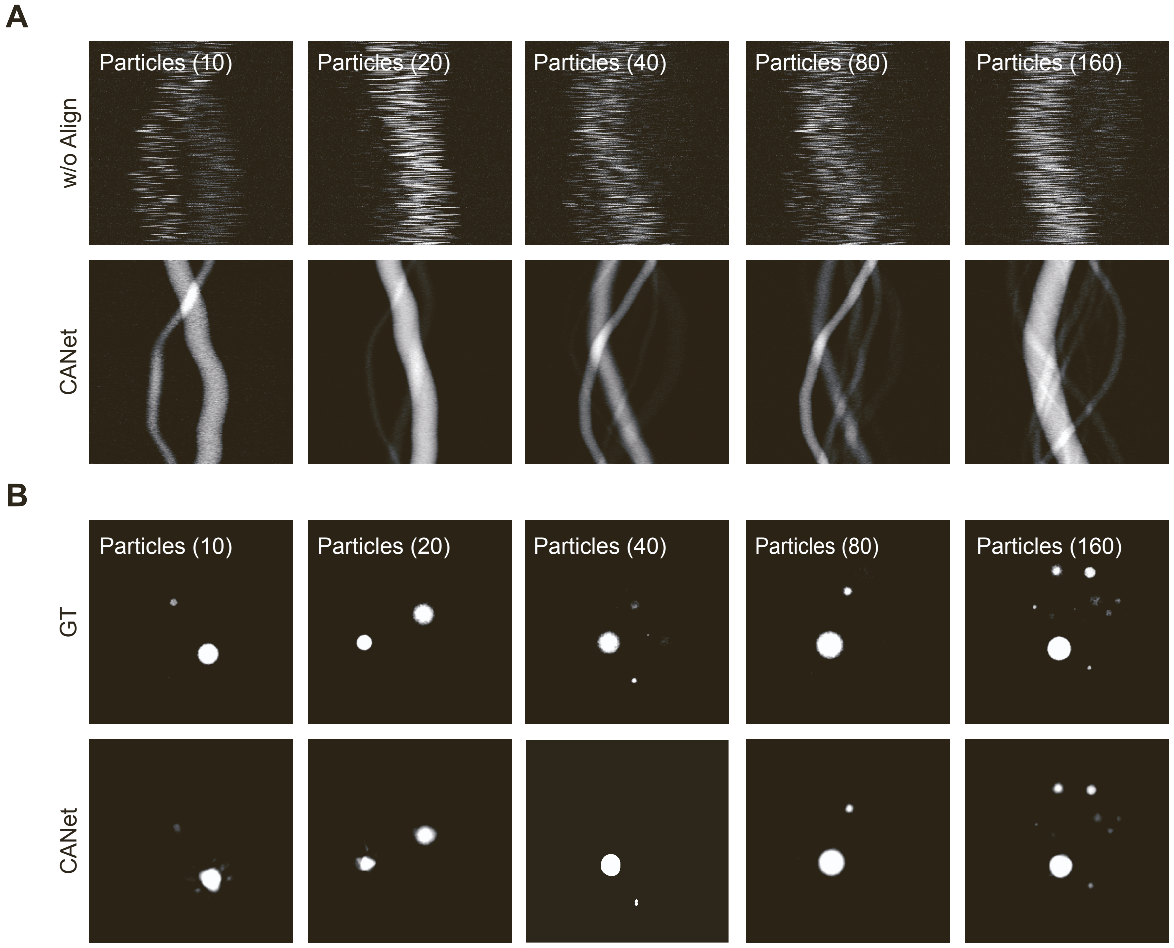

Figure S3. **Tomographic alignment performance with different particle counts.** (A) Representative sinograms of unaligned and CANet-aligned data. (B) Representative reconstructed slices of the ground truth and CANet-aligned.

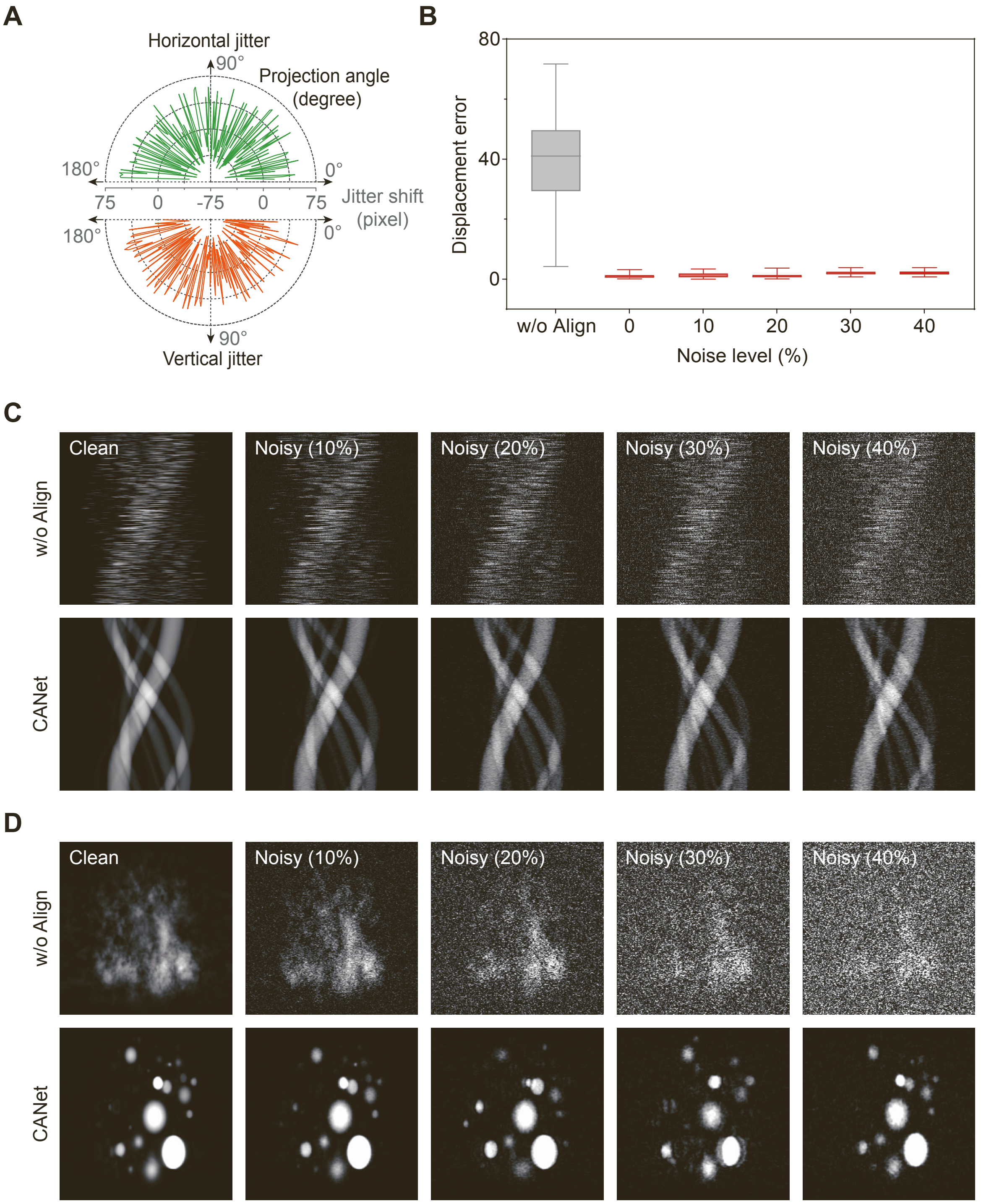

Figure S4. **Tomographic alignment performance under varying noise levels.** (A) Visualization of jitters in both horizontal and vertical directions across projection angles. (B) Quantitative displacement errors comparing between unaligned and CANet results across varying noise levels (0% to 40%). CANet achieves consistent high accuracy. (C)-(D) Representative sinograms and reconstructed slices of unaligned and CANet-aligned under noise-free and different noisy conditions.

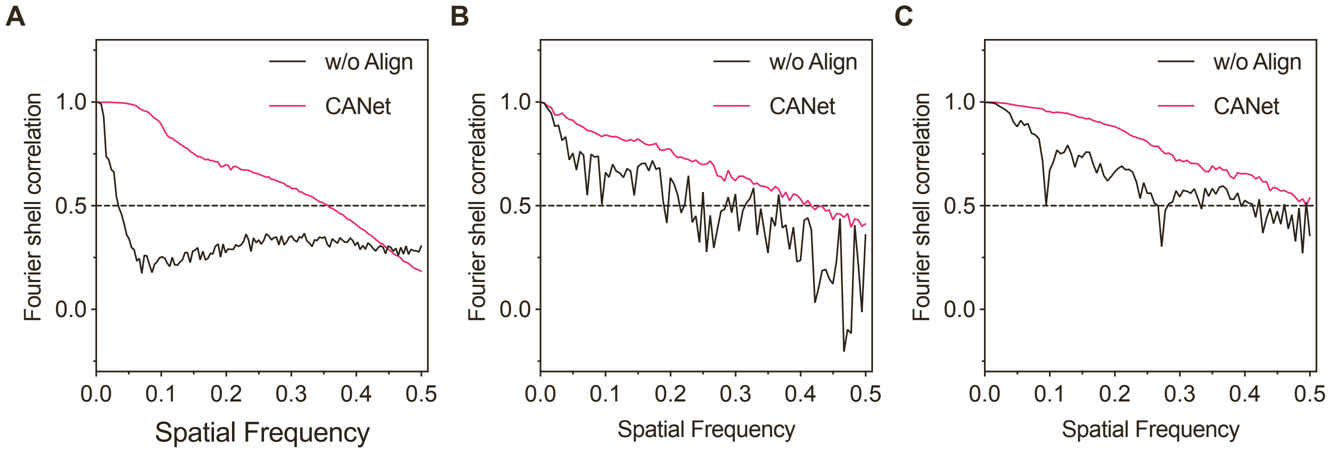

Figure S5. **Fourier shell correlation analysis.** (A) Simulation projection data in Figure 2. (B) The real NMC622 particle in Figure 4. (C) The Heterogeneous NMC particle in Figure 5.

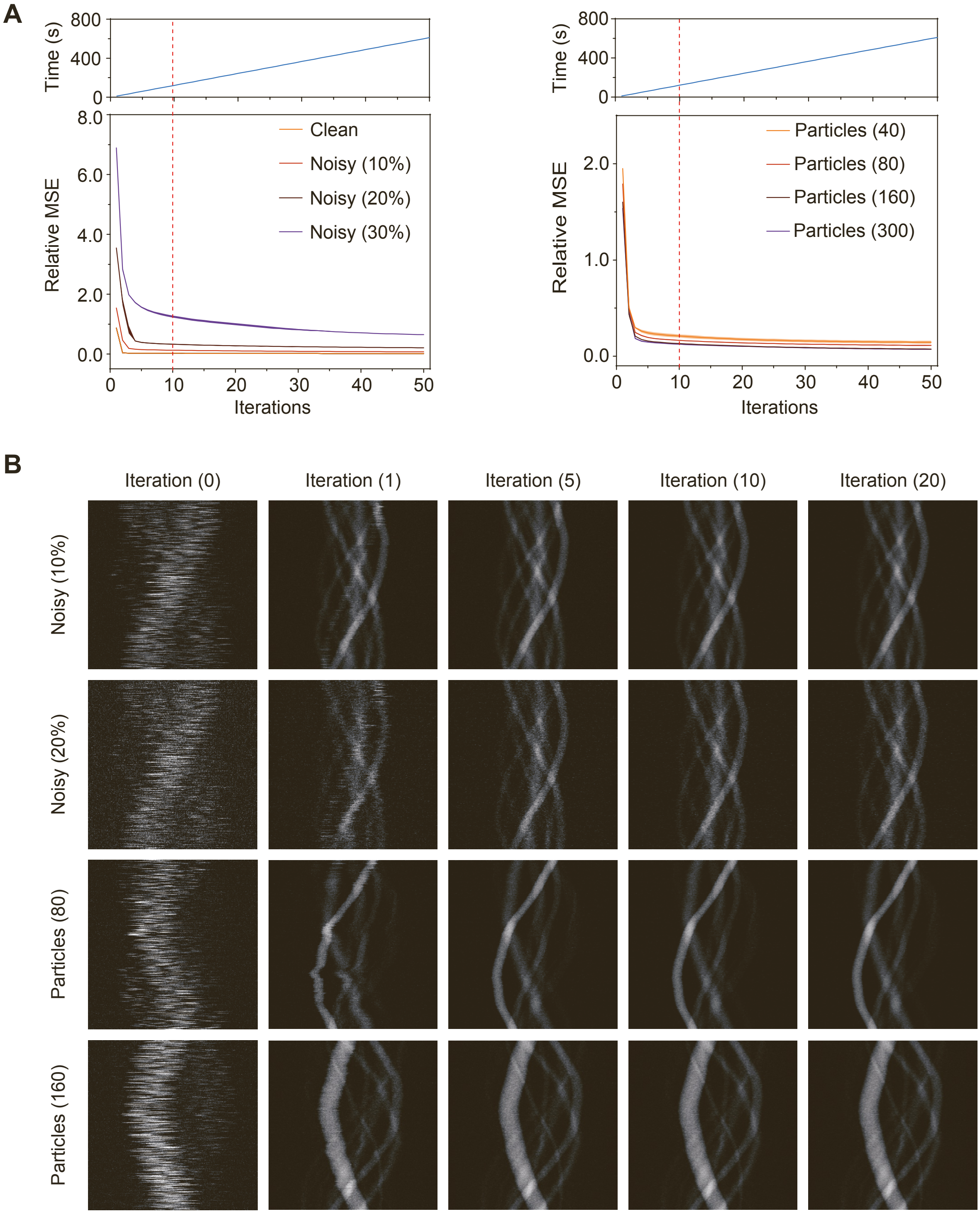

Figure S6. **Trade-off study between convergence efficiency and computational cost.** (A) Training dynamics showing running time and Relative MSE under varying noise levels and particle counts. The red dashed line (iteration 10) marks the optimal trade-off point, ensuring acceptable accuracy with minimized computation. (B) Representative sinograms at iterations 0, 1, 5, 10, and 20. Results show rapid stabilization by iteration 5, with minimal visual improvement observed thereafter, justifying the 10-iteration stopping point.

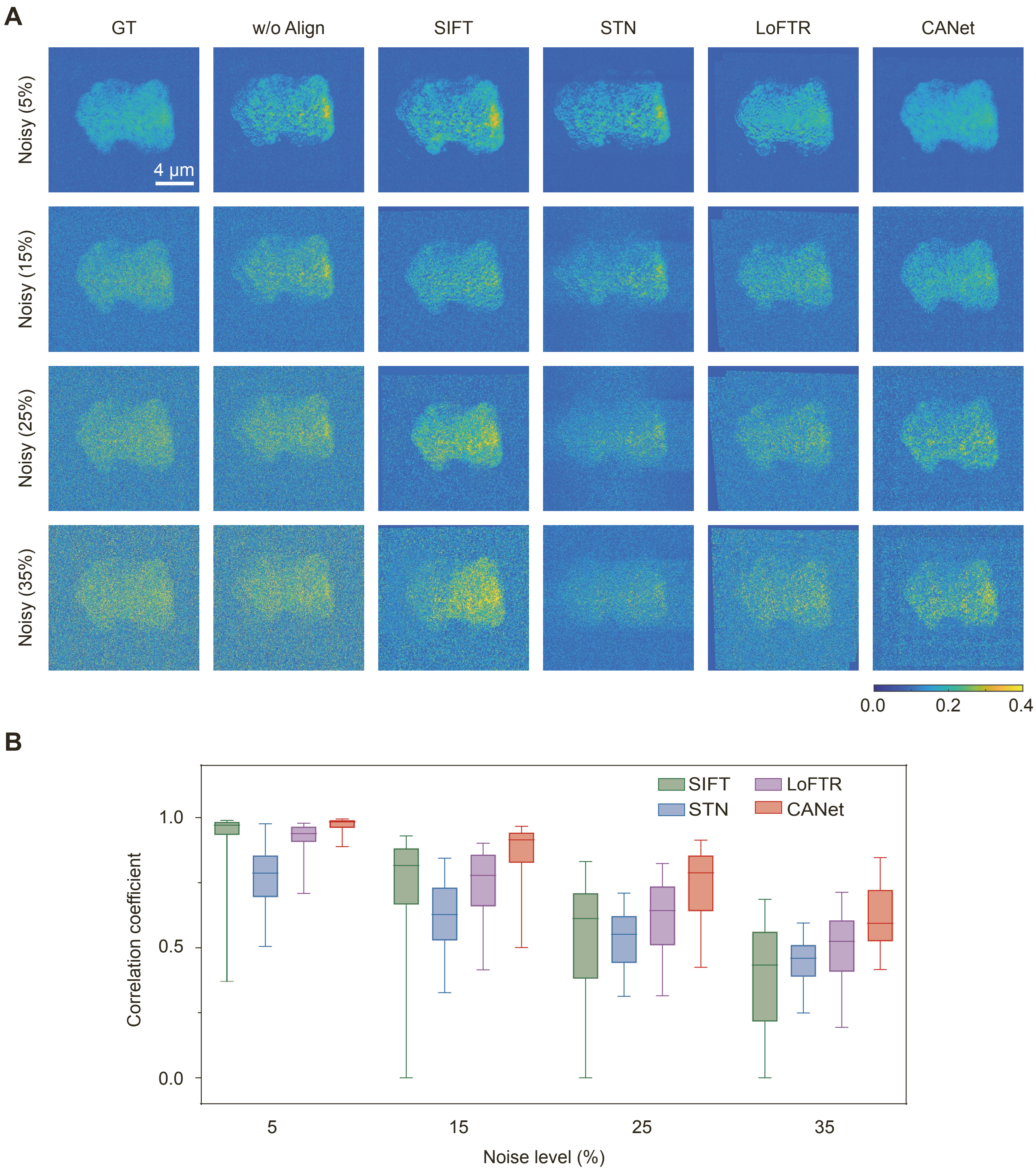

Figure S7. **Spectral alignment performance under varying noise levels (5% to 35%).** (A) Visual comparison of absolute residual errors for baselines and CANet. (B) Distribution of Pearson correlation coefficients under different noise conditions.

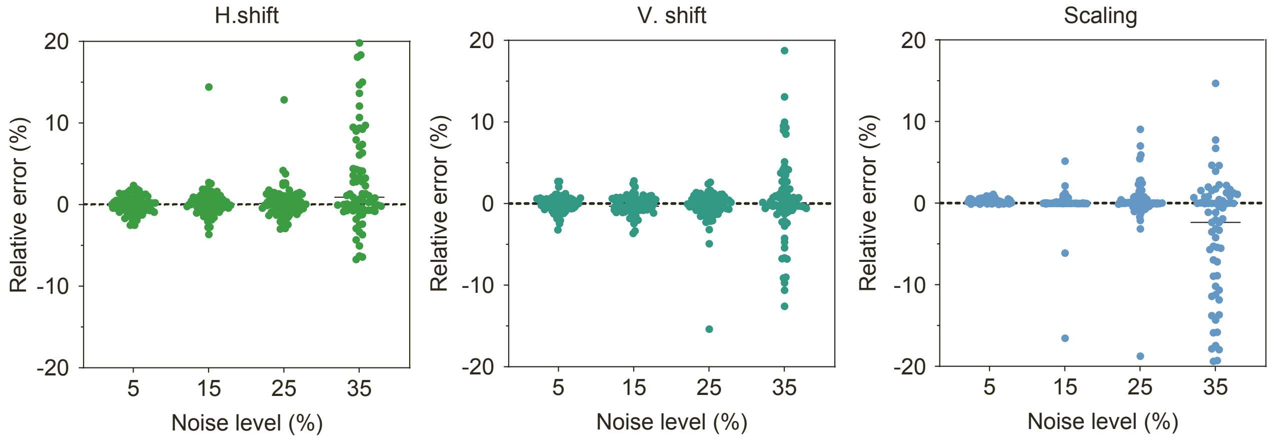

Figure S8. **Affine parameter estimations under noisy conditions for spectral alignment.** Relative errors for the horizontal shift (H. shift), vertical shift (V. shift), and scaling factor estimated by CANet.

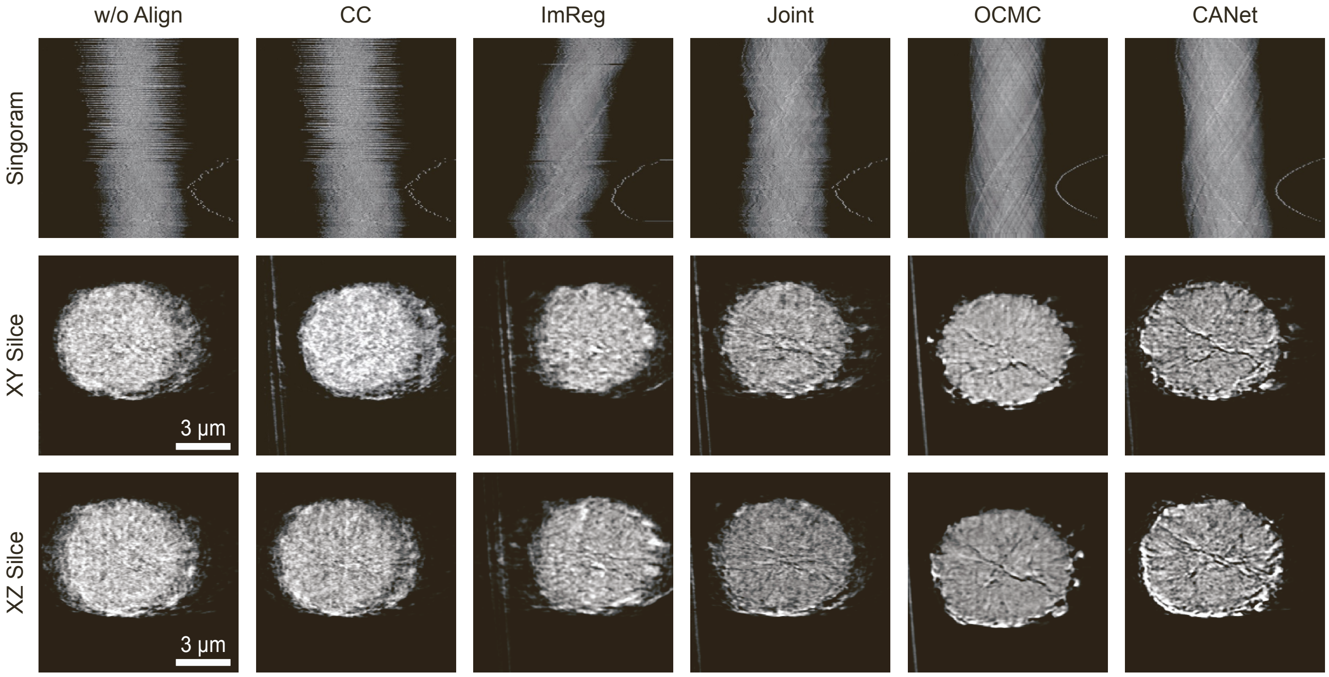

Figure S9. **Alignment performance of different methods for NMC622.** Representative sinograms (top), along with reconstructed 2D slices in the XY (middle) and XZ (bottom) planes.

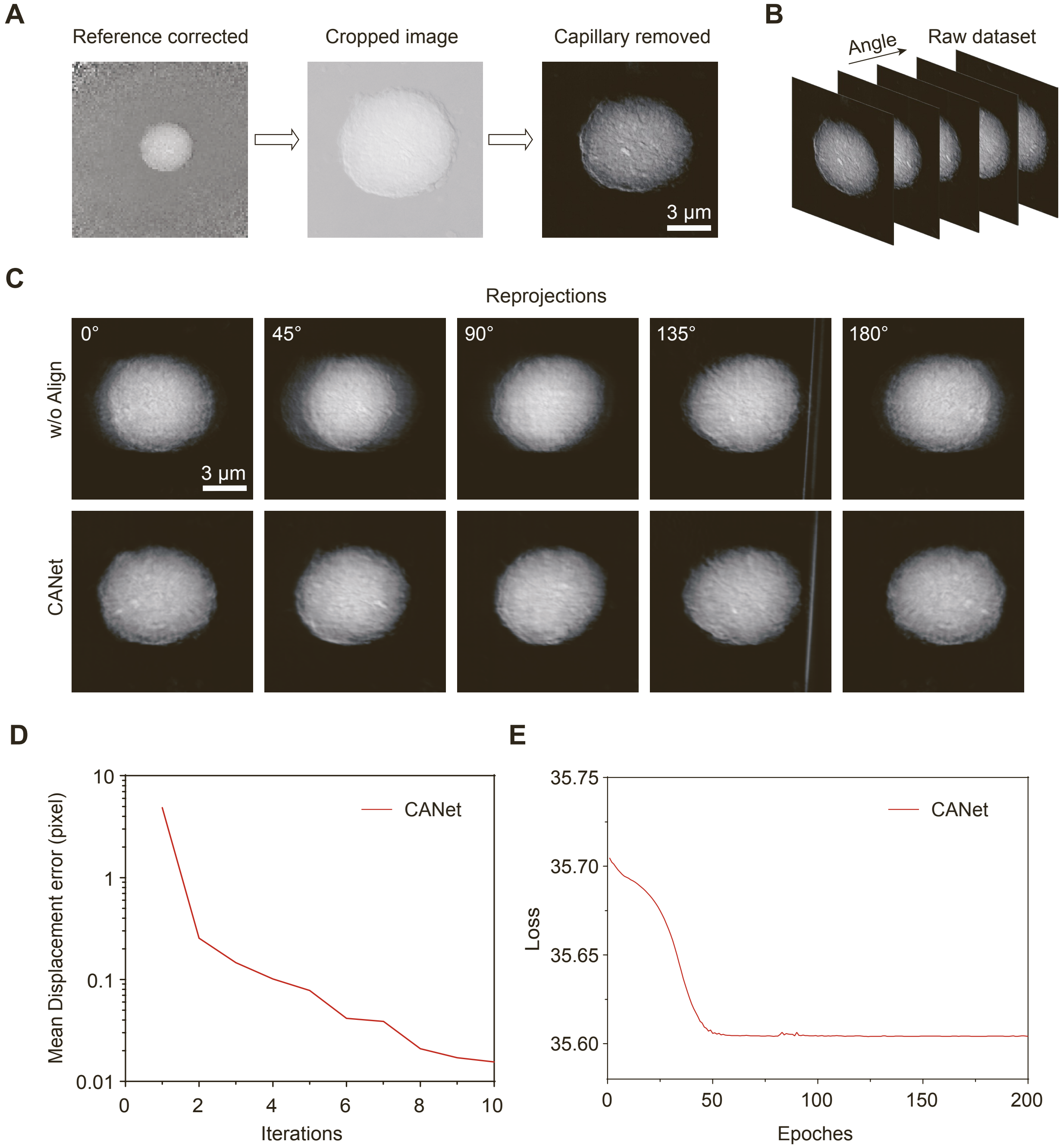

Figure S10. **Tomographic preprocessing and alignment for NMC622.** (A) Image preprocessing pipeline. (B) Raw projection data. (C) Representative reprojections across five angles between unaligned and CANet-aligned data. (D) Visualization of mean displacement error during projection-reprojection iterations. (E) Training loss trajectory over 200 epochs (1st iteration), the sharp decline at epoch 50 indicates a faster convergence.

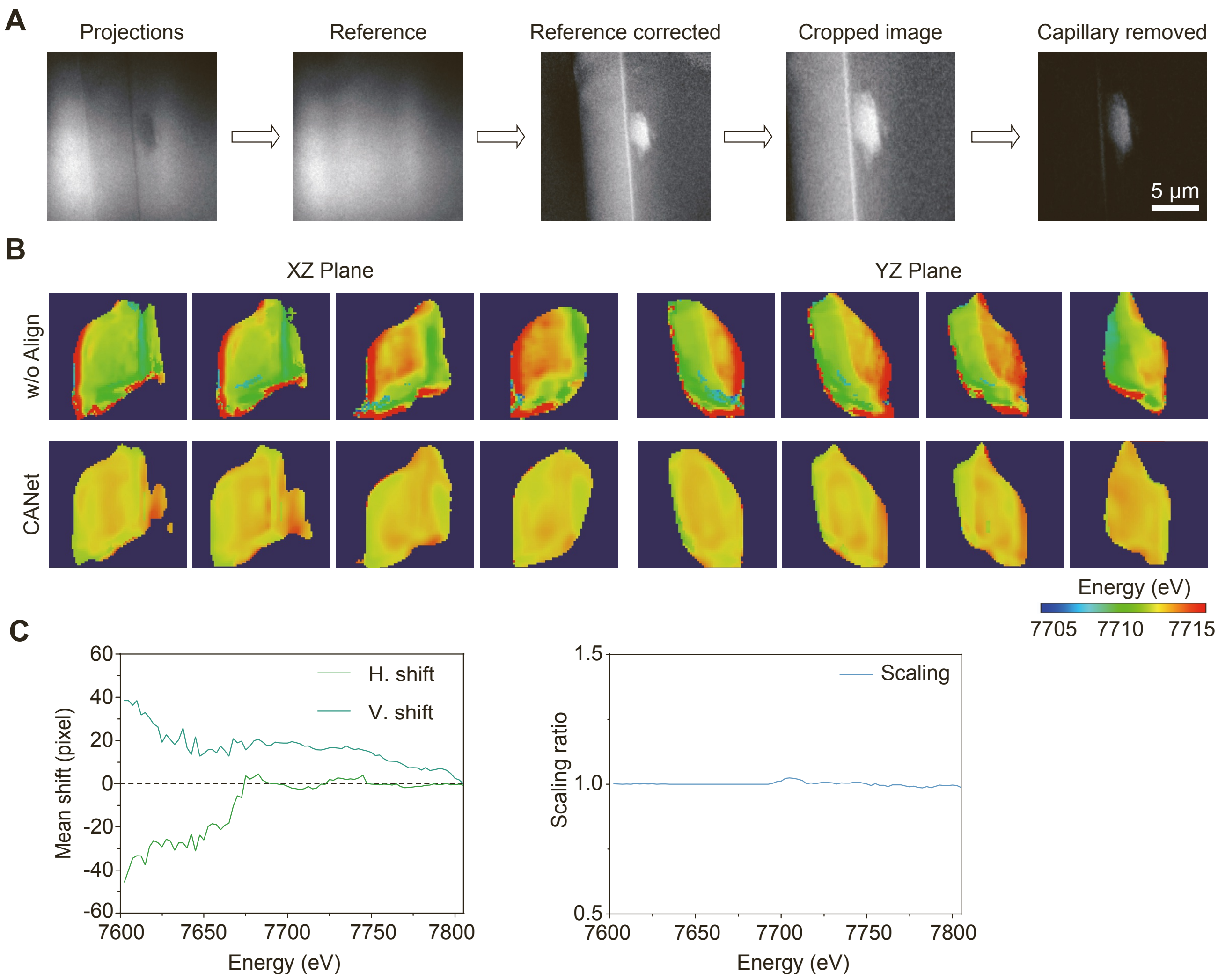

Figure S11. **Spectral preprocessing and alignment for LCO.** (A) Image preprocessing pipeline. (B) Representative reconstructed slices in the XZ and YZ planes, comparing unaligned and CANet-aligned data. (C) Estimation of average affine parameters (horizontal shift, vertical shift, and scaling) across all energy points.

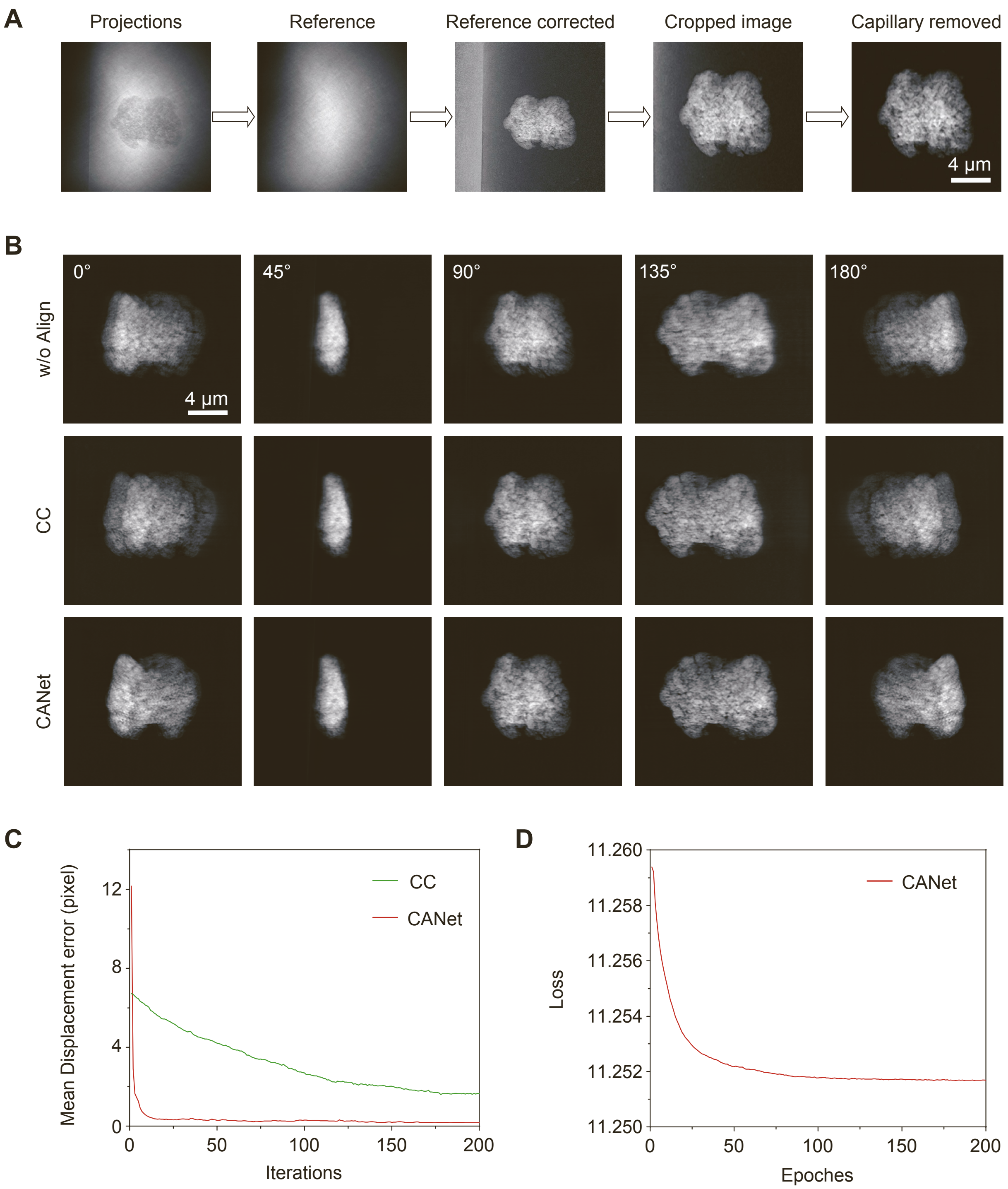

Figure S12. **Tomographic preprocessing and alignment for Heterogeneous NMC.** (A) Image preprocessing pipeline. (B) Representative reprojections comparing unaligned, CC-, and CANet-aligned results. (C) Mean displacement errors during the projection-reprojection iterations for CC and CANet. (D) Training loss trajectory of CANet across 200 epochs (1st iteration).

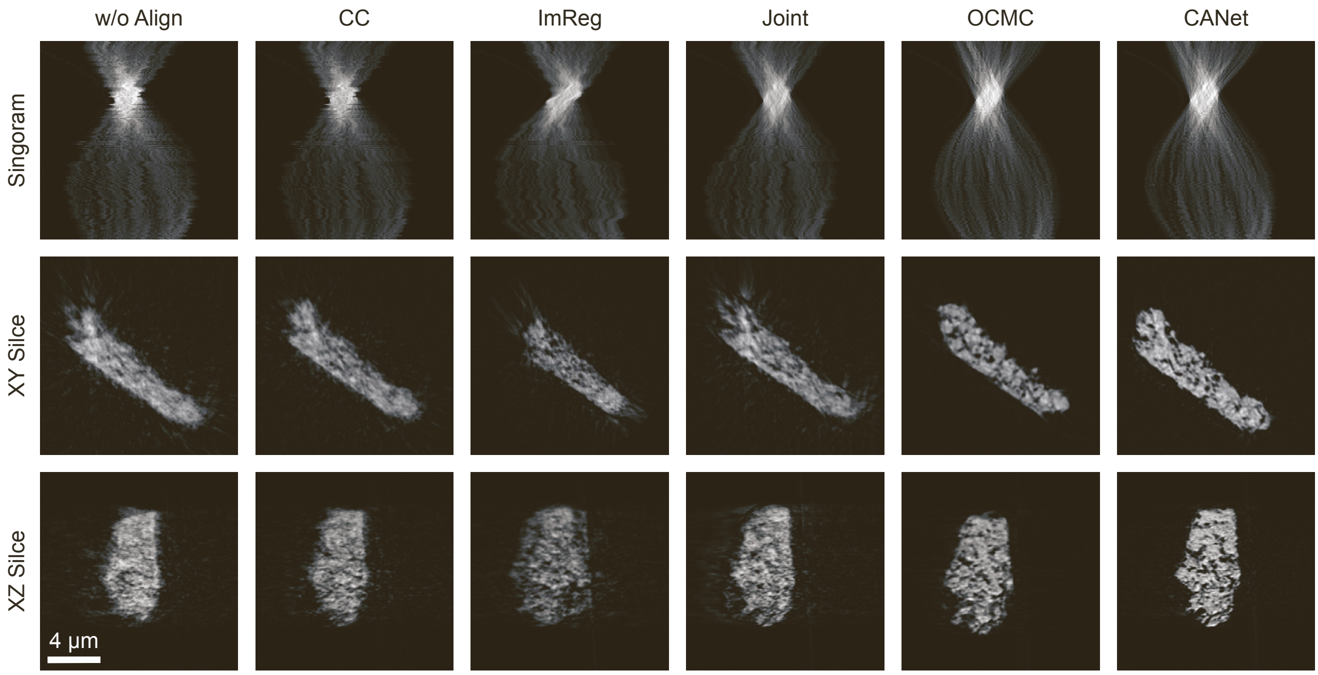

Figure S13. **Alignment performance of different methods on Heterogeneous NMC.** Representative sinograms (top), along with reconstructed 2D slices in the XY (middle) and XZ (bottom) planes.

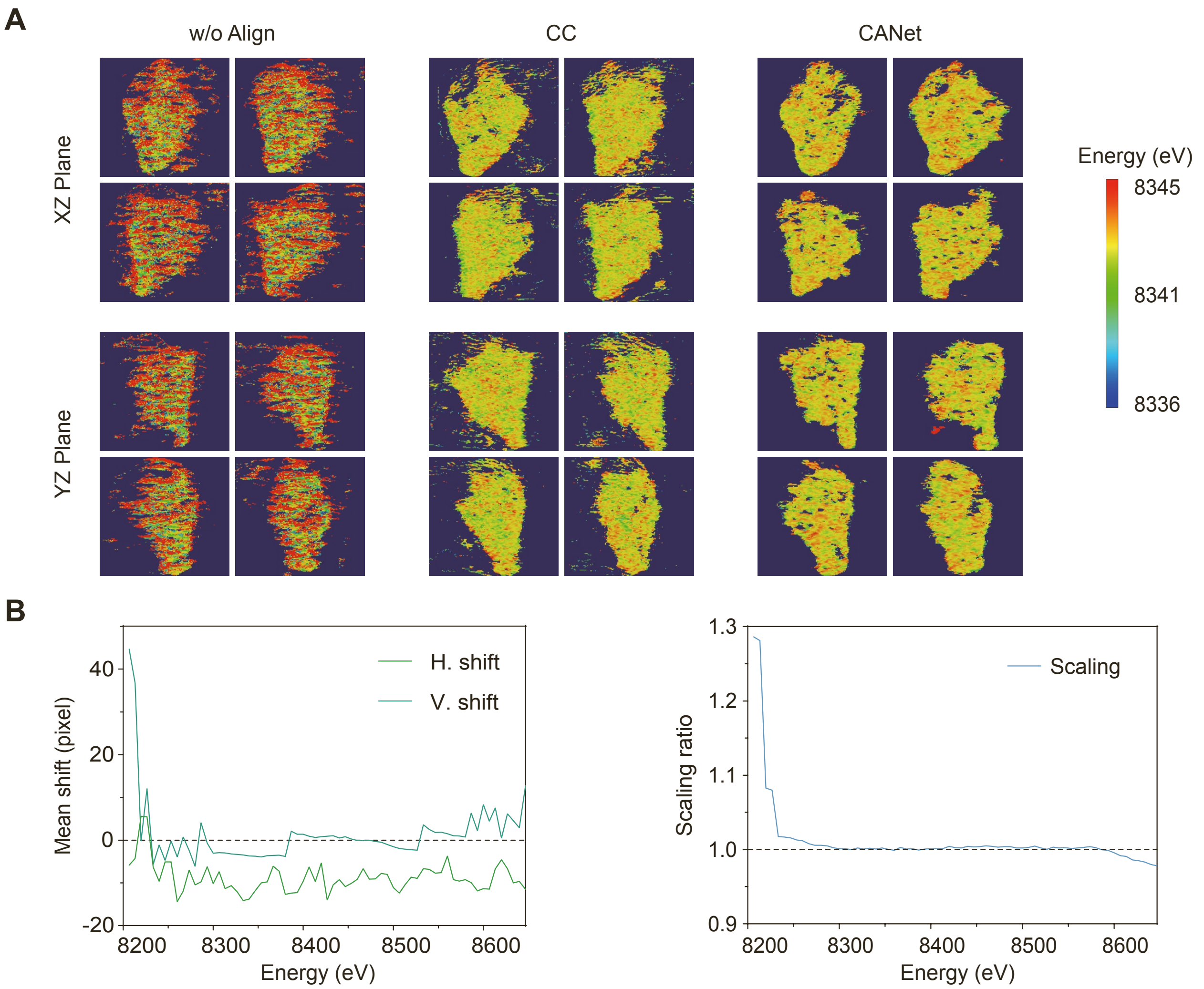

Figure S14. **Spectral preprocessing and alignment for Heterogeneous NMC.** (A) Representative 2D slices in XZ and YZ planes from unaligned, CC-, and CANet-aligned results. (B) The estimated average affine parameters of CANet across all energy points.

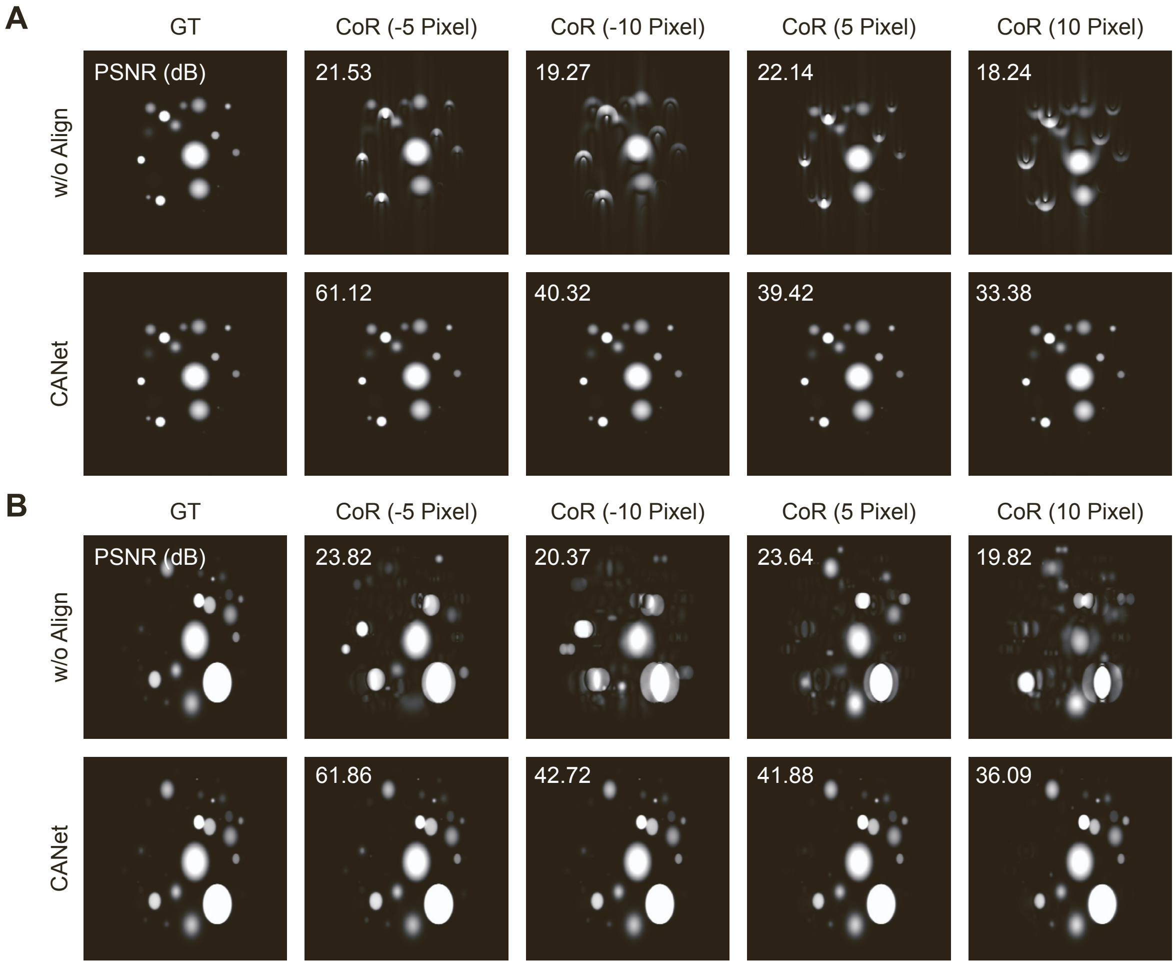

Figure S15. **Robustness against center of rotation offsets.** (A) Reconstructed slices in the XZ plane comparing unaligned (shifted by ±5 and ±10 pixels) with CANet-corrected data. (B) Corresponding comparisons in the XY plane.

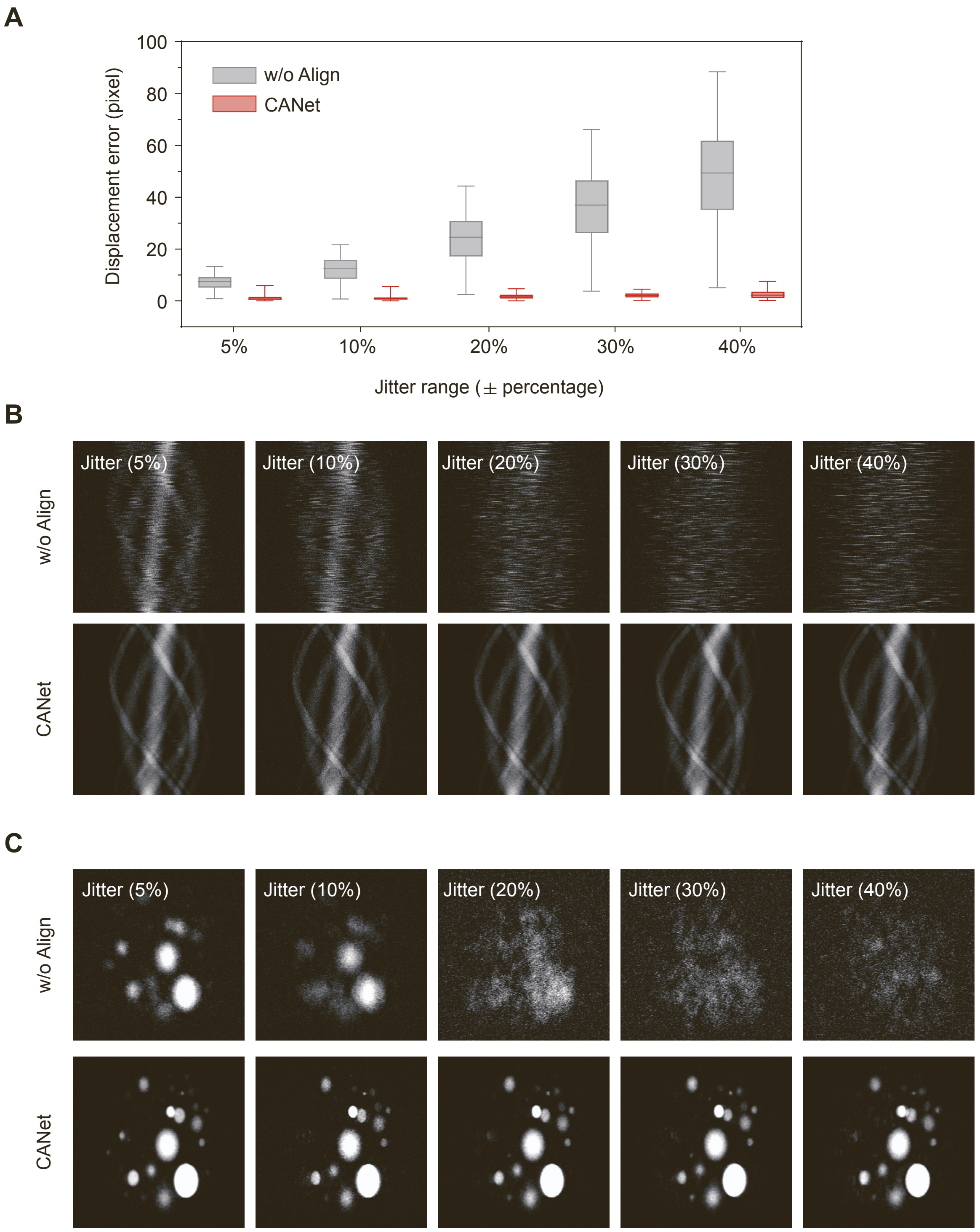

Figure S16. **Impact of jitter magnitude on alignment accuracy.** (A) Displacement errors for the unaligned baseline across different jitter magnitudes (5%-40%). (B)-(C) Representative sinograms and reconstructed slices of unaligned and CANet-aligned under different jitter magnitudes. The percentages indicate jitter magnitude relative to the maximum signal value.

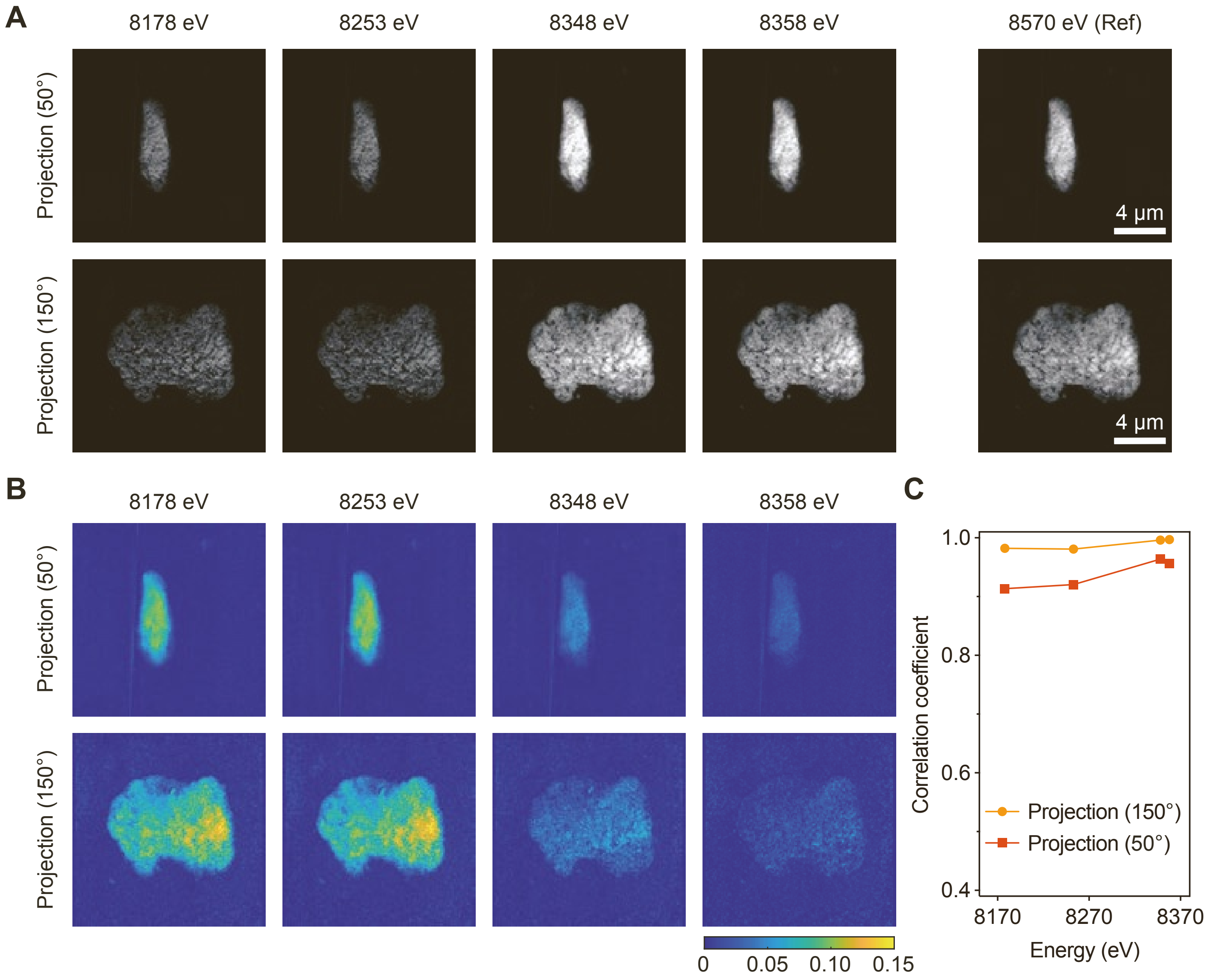

Figure S17. **Energy-dependent correlation analysis.** (A) Raw projections at 50°and 150°across different energies (8178eV, 8253eV, 8343eV, 8358eV, 8570eV (Ref)). (B) Absolute residual maps relative to the reference. (C) Correlation coefficients between each energy image and the reference.

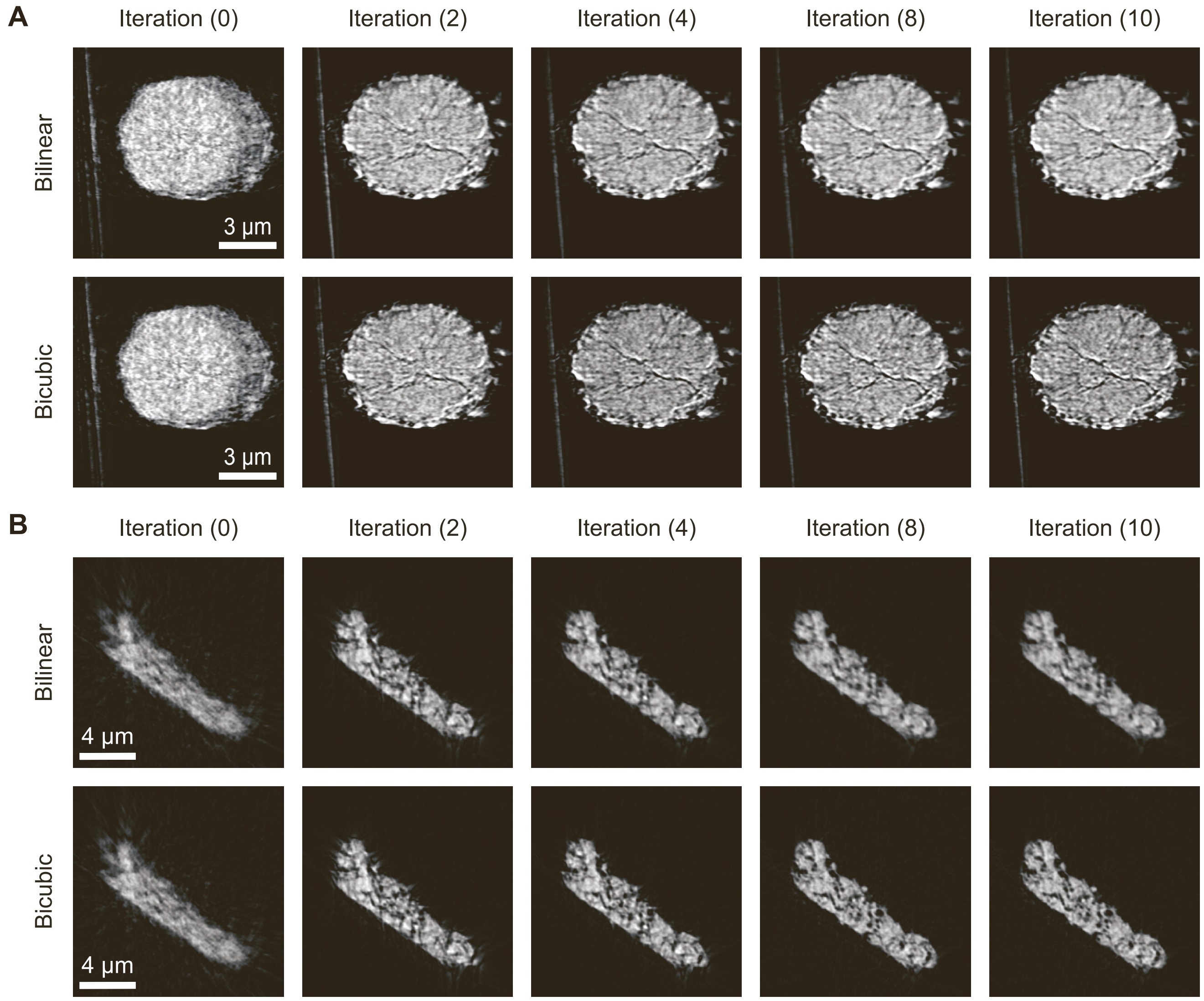

Figure S18. **Visual comparison between bilinear and bicubic interpolation methods.** (A)-(B) Representative reconstruction slices for Bilinear and Bicubic interpolation shown at iterations (0, 2, 4, 8, and 10).
